# Environmental determinants of infectious and chronic disease prevention behaviours: A systematic review and thematic synthesis of qualitative research

**DOI:** 10.1177/20551029231179157

**Published:** 2023-05-25

**Authors:** Abhinand Thaivalappil, Anit Bhattacharyya, Ian Young, Sydney Gosselin, David L Pearl, Andrew Papadopoulos

**Affiliations:** 1 Department of Population Medicine, 3653 University of Guelph, Guelph, Ontario, Canada; 2 School of Medicine, 6363 University of Ottawa, Ottawa, Ontario, Canada; 3 School of Occupational and Public Health, 7984 Ryerson University, Toronto, Ontario, Canada

**Keywords:** systematic review, health behaviour, qualitative research, COVID-19, smoking, health policy

## Abstract

Regulatory health policies facilitate desired health behaviours in communities, and among them, smoke-free policies and COVID-19 restrictions have been widely implemented. Qualitative research studies have explored how these measures and other environmental influences shape preventive behaviours. The objective of this systematic review was to synthesize previously published qualitative research, generate across-study themes, and propose recommendations for behaviour change interventions. We used a comprehensive search strategy, relevance screening and confirmation, data extraction, quality assessment, thematic synthesis, and quality-of-evidence assessment. In total, 87 relevant studies were identified. Findings were grouped under six overarching themes and mapped under three categories: (i) the political environment, (ii) the sociocultural environment, and (iii) the physical environment. These findings provide insights into the environmental influences of behaviour and indicate future interventions may be more effective by considering moral norms, community norms, policy support, and group identity.

## Introduction

The global burden of disease can be reduced through the modification of behavioural risk factors (Abbafati et al., 2020; Linardakis et al., 2015). However, an analysis of globally aggregated data from 2019 reported there was no meaningful progress in reducing exposures to behavioural risks between 1990–2019 (Abbafati et al., 2020) which indicates current interventions and approaches may not be sufficient in achieving long-term, sustainable health behaviour change. This has led to increased support and recommendations for regulatory health policies as an effective public health intervention when other strategies such as educational interventions and community programmes fail (Abbafati et al., 2020; Brown et al., 2014; Frazer et al., 2016; Hillier-Brown et al., 2017; Thomas et al., 2008). In 1986, the Ottawa Charter for Health Promotion advocated for the socioecological model, which proposes two concepts: (i) behaviour both shapes and is shaped by multiple levels of influence; and (ii) individual behaviour affects and is affected by the social environment (World Health Organization, 2022). Health models which included the ecological component were early advocates for community wellbeing, which emphasized factors in the environment - such as community, neighbourhood, cultural norms, and policies - rather than modifying individual habits and behaviours. This also acknowledged the existence of complex individual-environmental interactions across all levels of behaviour (Golden and Earp, 2012; McLeroy et al., 1988; Stokols, 1992). As a result, people were viewed within the context of their environments, and health promotion programs highlighted solutions to modify these conditions to promote favourable health behaviours (Golden and Earp, 2012).

The success of environmental interventions has been demonstrated previously in the implementation of regulatory smoke-free policies and more recently in the novel coronavirus disease 2019 (COVID-19) government restrictions. Although tobacco use is still prevalent globally, public health approaches to tobacco interventions in the form of regulatory health policies have reduced smoking, reduced second-hand smoke exposure, and improved health outcomes over time in many regions (Centers for Disease Control and Prevention, 2020; Linardakis et al., 2015; World Health Organization, 2014). COVID-19 was also a major worldwide public health burden which led to the swift introduction of regulatory policies promoting prevention and control, such as stay-at-home orders, physical distancing, and mandatory mask use in community settings (Gebru et al., 2020). In the absence of pharmaceutical interventions for much of 2020, government restrictions reduced COVID-19 transmission (Brauner et al., 2021; Haug et al., 2020; Wibbens et al., 2020). Particularly, more intrusive regulations which focus on movement restriction such as workplace and school closures, stay-at-home orders, and travel bans reduced infection rates (Haug et al., 2020; Wibbens et al., 2020).

Through this, it is evident the implementation of regulatory policies drives behaviour change through increased compliance. However, downstream impacts and between-level relationships with other environmental (e.g., built environment, culture, community), social, and individual factors of disease prevention behaviours are underexplored. Qualitative research exploring smoke-free policies and COVID-19 restrictions have investigated public perceptions and beliefs surrounding impacts of such factors on preventive health behaviours (Chen et al., 2021; Hackshaw et al., 2012; Heid et al., 2021; Highet et al., 2011). The outcomes of this review will provide insights into the environmental influences of behaviour, reveal relationships between levels of behaviour, and offer guidance for future policy implementation and behaviour change interventions.

The purpose of this study was to conduct a systematic review and thematic synthesis of qualitative primary research investigating the environmental determinants of smoke-free policies and COVID-19 restrictions on individuals' infectious and chronic disease prevention behaviours to generate overarching themes and novel interpretations of results (Mays et al., 2005; Thomas and Harden, 2008). The literature on smoke-free policies and COVID-19 restrictions were combined as both: (a) were under the direct influence of regulatory policies at the time of this review, (b) emphasize preventive actions in community settings to protect health, and (c) have a substantial amount of published primary qualitative literature. No study has used structured and transparent knowledge synthesis methods to identify, characterize, and synthesize themes from all available qualitative studies in this area.

## Method

### Review approach

This review followed the Cochrane Collaboration handbook (Higgins et al., 2021), and thematic synthesis guidelines outlined by Thomas & Harden (Thomas and Harden, 2008). A reporting guideline for qualitative research syntheses, known as the Enhancing transparency in reporting the synthesis of qualitative research (ENTREQ) framework was also followed (Tong et al., 2012). The review question was: “What are the cultural, societal, and regulatory policy determinants on individuals’ infectious and chronic disease prevention attitudes, beliefs, and behaviours?” The population and setting of interest were adults aged 18 or older in a community setting. Studies were excluded if they investigated preventive health behaviours in the private setting such as the home and multiunit housing; employees in the organizational context (e.g., healthcare staff, restaurant workers); institutionalized individuals (e.g., patients, prisoners); and adolescents and children <18 years old. The outcome of interest was behavioural determinants (e.g., attitudes, beliefs, intentions) and behaviour. Studies were included only if they were conducted in countries classified by the United Nations Development Programme as “very high human development” because outcomes could be more applicable to North American policymakers and public health practitioners (United Nations Development Programme, 2019). Qualitative and mixed methods primary research studies published in English, French, or Spanish in any year were considered for inclusion. In addition to peer-reviewed journal articles, selected grey literature such as theses, conference proceedings, research reports, and government reports were selected for inclusion based on recommendations outlined in the Cochrane Handbook for Systematic Reviews of Interventions (Higgins et al., 2021). A review protocol outlining the proposed methodology was established prior to the conduct of the review. A copy of this review protocol, ENTREQ checklist, and search documentation can be accessed as supplementary material.

### Search strategy

A comprehensive search strategy was developed and pre-tested. Following this, a full search was conducted on 31 October 2020 and later updated on 19 December 2022 using the following databases: Sociological Abstracts, Applied Social Sciences Index & Abstracts, ProQuest Dissertations and & Theses A&I, PsycINFO, Web of Science Core Collection, and PubMed. Search categories consisted of the topic of interest (e.g., COVID-19, smoke-free, tobacco-free), population of interest (e.g., adult, customer, community member), exposure terms (e.g., restriction, policy, ban, regulation, culture, societal norm), outcome terms (e.g., knowledge, attitude, belief, behaviour), and study type (e.g., qualitative, focus group, mixed methods).

Gaps may exist in database indexing of qualitative research which result in some relevant studies being missed (Shaw et al., 2004). To supplement the search strategy, additional searches for potentially missed articles and grey literature were conducted through Google and Google Scholar using a combination of search terms (Canadian Agency for Drugs and Technologies in Health, 2015; Haddaway et al., 2015). The first 100 hits of each search were examined for relevance for practical reasons (Canadian Agency for Drugs and Technologies in Health, 2015) with a total of six search strings and 1200 hits being inspected. Lastly, reference lists of a few notable relevant studies were examined to pull out any potentially missed reports or publications not captured by the database searches. Detailed information on the search algorithm and parameters used for each database are provided as supplementary material.

### Relevance screening, data extraction, and quality assessment

Article titles and abstracts were assessed for relevance by AT and AB using a structured screening form. Full texts of relevant references were obtained, confirmed for relevance (AT and AB) and study characteristics were extracted (completed by AT, AB, and SG) using a second structured characterization form. This form captured general characteristics (e.g., year published, country), details on study design, methodology, mode of conduct, participant recruitment, policy focus of the study, sample size, and details on the study population.

This structured form also contained a quality assessment tool which was previously developed and implemented in other systematic reviews of qualitative research (Thaivalappil et al., 2018; Walsh and Downe, 2006; Young and Waddell, 2016). The tool was used to critically appraise and determine the transparency, integrity, and limitations of qualitative research studies in this review (Walsh and Downe, 2006). Quality assessments provide insights into the trustworthiness of qualitative research studies (Stenfors et al., 2020). The tool contained eight criteria which assesses whether study authors report the following in sufficient detail: study objectives; study design; sampling strategy; analytic approach; findings and interpretations; researcher reflexivity (i.e., providing context on the effect of the researcher on the participants); ethical considerations; and limitations, implications and transferability (Walsh and Downe, 2006). The inputs from these criteria are summated to assess whether any gaps exist in reporting and inform recommendations for future research. Screening, data extraction, and quality assessment were completed by a minimum of two independent reviewers (AT, AB, and SG) and any conflicts that arose were resolved through discussion. The results of these are provided in the supplementary material.

### Review management

All identified references were uploaded to the reference management software Mendeley (Elsevier Inc., New York, NY) and de-duplicated before being imported to a spreadsheet (Excel, Microsoft Office 365, Microsoft Corporation, Redmond, WA) where relevance screening, confirmation, data extraction, and quality assessment were conducted. The relevance screening tool was pre-tested on 30 abstracts whereas other forms were pre-tested on five articles. An inter-rater reliability kappa agreement of >0.80 was met before proceeding with relevance screening. For other review forms, minor modifications were applied to meet consistent reviewer interpretation of questions. All stages of the review except for the Grading of Recommendations Assessment, Development, and Evaluation - Confidence in the Evidence from Reviews of Qualitative research (GRADE-CERQual) assessments were completed by two independent reviewers (AT and AB). Any reviewer disagreements were minor in nature and were resolved through discussion and consensus after each review stage. A content and systematic review expert from the research team was consulted for clarification and resolution of any unsettled disagreements between reviewers. Copies of all review forms are available in the supplemental files.

### Data analysis

A modified thematic synthesis approach was followed for qualitative analysis (Thomas and Harden, 2008). This approach was followed because it extends beyond the content of the primary qualitative research studies to form new interpretations and generate themes in a transparent manner (Thomas and Harden, 2008). The synthesis involves three stages: (a) line-by-line coding of participants' and study authors' experiences, (b) organization of these codes into descriptive themes, and (c) from this, ‘analytical’ themes, sometimes referred to as third-order interpretations, were generated by one reviewer and verified by the second reviewer (Thomas and Harden, 2008). Third-order interpretations are described as when the researcher goes beyond the content of the original studies to answer the review question (Thomas and Harden, 2008).

The coding framework was generated inductively, where articles with conceptually rich information were selected as a basis for the development of the codebook. Two members of the research team (AT and AB) generated codes with no assumptions on how they should be defined or structured. These initial codes were iteratively compared across studies to ensure they fit the criteria for societal determinants of preventive health behaviours. A reading of all results from the relevant articles were completed by AT and AB until all codes were captured. Next, codes were mapped to environmental factors outlined in the ecological model of health behaviour and the social ecology model for health promotion; both models state that factors beyond the intrapersonal level influence health behaviour (McLeroy et al., 1988; Stokols, 1992). Results sections of relevant articles were imported to NVivo 1.7.1 qualitative analysis software (QSR International, Doncaster, Australia) where coding was then conducted. The coding framework is available as supplementary material.

Although no articles were excluded based on within-study quality assessment rating, we went beyond the quality assessment, and incorporated into GRADE-CERQual to identify the level of confidence in each review finding (Lewin, Booth, et al., 2018). The GRADE-CERQual approach is similar to quantitative syntheses where the purpose is to provide the reader with additional information on how much confidence to place in review findings for qualitative syntheses (Lewin, Booth, et al., 2018). In this case, a review finding is defined as an output that explains a phenomenon or an aspect of it (Lewin, Booth, et al., 2018), and thus, we considered each subtheme as an independent review finding. CERQual is based on four criteria: adequacy of data, relevance, coherence, and methodological limitations. Adequacy of data describes the richness and quantity of data; methodological limitations describes potential concerns about study design or conduct and is extrapolated from the quality assessment; coherence assesses the degree of consistency of findings across studies; and relevance describes how well the body of evidence from the primary qualitative research supports the review finding and whether there were potential variations in the setting or population (Lewin, Booth, et al., 2018). Criteria were rated as having no concerns, minor concerns, moderate concerns, or substantial concerns. An overall confidence rating was determined based on individual ratings of the criteria and was rated as having high confidence (i.e., it is highly likely that the finding is a reasonable representation of the phenomenon of interest), moderate confidence (i.e., it is likely that the finding is a reasonable representation of the phenomenon of interest), or low confidence (i.e., it is unclear whether the finding is a reasonable representation of the phenomenon of interest) (Lewin, Booth, et al., 2018). The original CERQual approach contains four overall ratings: high confidence, moderate confidence, low confidence, and very low confidence. However, the latter two categories were combined as we found little to no variability between them, similar to previous syntheses (Thaivalappil et al., 2018; Young and Waddell, 2016). The first author (AT) conducted a preliminary CERQual assessment which was reviewed and validated by a second team member (AB). Minor changes were made to the individual and overall ratings after discussion between both reviewers. A copy of the GRADE-CERQual assessment and studies supporting each review finding is accessible as supplementary material.

## Results

### Study characteristics

**Figure 1.**
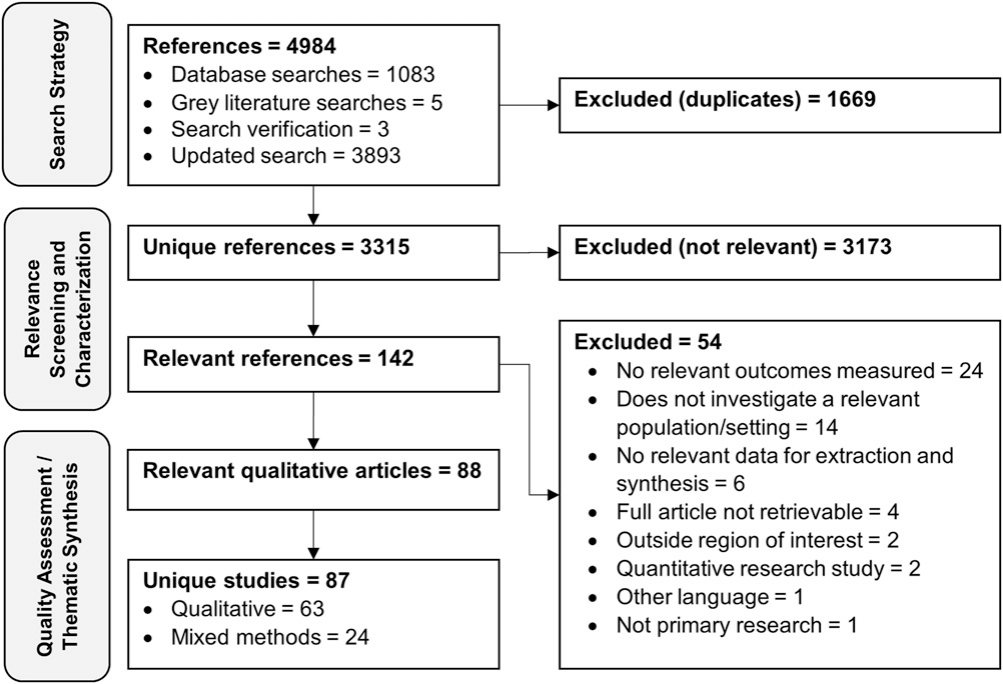
Flow diagram of the systematic review process.

Overall, 88 relevant articles were identified representing 87 unique studies and percentages were calculated using the former as the denominator. The discrepancy in numbers were due to a thesis and a published article by the same author containing similar data, with the former reporting more qualitative results. A flow diagram of the systematic review process is shown in Figure 1 and a summary of the descriptive study characteristics of the relevant articles and studies is shown in Table 1. Most articles captured COVID-19 rather than smoke-free policies (*n* = 50, 56.8%), were qualitative (vs. mixed methods) (*n* = 64, 72.7%), did not specify the qualitative methodology used (*n* = 65, 73.9%), were based out of the United States (US) and United Kingdom (UK) (*n* = 46, 52.3%), and used one-to-one interviews for data collection (*n* = 55, 62.5%). The median publication year of smoke-free articles was 2011 (range 2006–2020) and overall relevant articles was 2020 (range 2006–2022). The median sample size of relevant studies was 28 (range 7–2081). Studies which conducted one-to-one interviews had a median of 25 interviews (range 1–208) with two studies not reporting the number of interviews. Among studies that conducted focus groups (*n* = 26, 29.5%), the median number of focus groups conducted per study was 7 (range 1–29) with three studies not reporting sampling details. Of the studies which recorded open-ended responses from questionnaires (*n* = 17, 19.3%), only nine reported the number of responses used for qualitative analysis.

**Table 1.** Study characteristics of the 88 qualitative and mixed methods articles investigating smoke-free policies and COVID-19 restrictions identified in this systematic review.

Characteristic	N	%
Document type		
Journal article	84	95.5
Report	2	2.3
Thesis	2	2.3
Country^a^		
United States	25	28.4
United Kingdom	21	23.9
Canada	10	11.4
Australia	4	4.5
Belgium	3	3.4
Greece	2	2.3
Ireland	2	2.3
Japan	2	2.3
Malaysia	2	2.3
Singapore	2	2.3
Spain	2	2.3
Sweden	2	2.3
Switzerland	2	2.3
Other^b^	14	15.9
Language		
English	87	98.9
Spanish	1	1.1
Qualitative methodology^a^		
Grounded theory	5	5.7
Phenomenology	5	5.7
Ethnography	4	4.5
Qualitative descriptive	4	4.5
Case study	3	3.4
Community-based participatory research	2	2.3
Basic interpretive	1	73.9
Not specified	65	5.7
Data collection methods^a^		
Interviews	55	62.5
Focus groups	26	29.5
Questionnaire	17	19.3
Observation	1	1.1
Online discussion posts	1	1.1
Not specified	1	1.1
Data were collected on more than one occasion		
Yes	18	20.5
No	70	79.5
Mode of conduct specified		
Yes	71	80.7
No	17	19.3
Method of participant recruitment specified		
Yes	77	87.5
No	11	12.5
Specific sociodemographic group focus^a^		
Smokers and ex-smokers	20	22.7
Older adults	13	14.8
Ethnocultural group^c^	13	14.8
Students and young adults	9	10.2
Parents	6	6.8
Low socioeconomic status	4	4.5
Women	3	3.4
LGBT	2	2.3
Other^d^	6	6.8
None	27	30.7
Smoke-free policy focus (*n* = 38)^a,e^		
Restaurants, bars, pubs, and nightclubs	9	23.7
Public areas	5	13.2
Bingo halls, and casinos	2	5.3
Sporting venue	1	2.6
None	23	60.5
COVID-19 policy focus (*n* = 50)^a,e^		
Lockdown, stay-at-home, or quarantine	20	40.0
Physical distancing	14	28.0
Face masks	8	16.0
Age-based recommendations (70+)	1	2.0
None	19	38.0
Quality assessment criteria met by each article (yes vs. no)		
Clear statement of research aims	87	98.9
Research design and data collection strategy clearly described and appropriate to address the research aims	83	94.3
Sampling strategy clearly described and appropriate to address the research aims	74	84.1
Method of analysis clearly described and appropriate to address the research aims	65	73.9
Findings clearly described and supported by sufficient evidence	86	97.7
Evidence of researcher reflexivity	28	31.8
Ethical issues taken into consideration	63	71.6
Evidence of study relevance and transferability	77	87.5

^a^Some articles had multiple selections for this category and percentages may exceed 100%.

^b^One study was found across the following countries: Argentina, Austria, Denmark, Finland, Germany, Israel, Norway, Saudi Arabia, Scotland, Serbia, Slovenia, South Korea, The Netherlands, Turkey, and Uruguay.

^c^Groups included African American (*n* = 1), Bangladeshi (*n* = 1), Chinese (*n* = 4), Indigenous (*n* = 2), Irish (*n* = 1), Jewish (*n* = 1), Latino (*n* = 1), and Muslims (*n* = 2).

^d^One of each: couples living together, households with at least one positive COVID-19 case, migrants, soldiers, people with asthma, and tourists/hotel residents.

^e^Percentages were calculated using this value as the denominator.

Most studies in this review targeted specific groups of people for investigation (*n* = 61, 69.3%). Among them, the most frequently investigated were smokers and ex-smokers (*n* = 20, 22.7%), older adults (*n* = 13, 14.8%), and ethnocultural groups (*n* = 13, 14.8%). Among studies which investigated smoke-free policies (*n* = 38), those that indicated a focused setting frequently discussed restaurants, bars, pubs, and nightclubs (*n* = 9, 23.7%) as opposed to public spaces such as parks and beaches (*n* = 5, 13.2%) and recreational venues (casino, bingo hall, hockey arena) (*n* = 3, 7.9%). Studies which investigated COVID-19 policies (*n* = 50) commonly investigated lockdown or stay-at-home orders (*n* = 20, 40.0%) and physical distancing (*n* = 14, 28.0%).

Key criteria that reduced study quality were the following: evidence of researcher reflexivity (*n* = 28, 31.8%), details on ethical considerations (*n* = 63, 71.6%) and qualitative analysis used (*n* = 65, 73.9%). More information on study characteristics details, quality assessment ratings, and a citation list of relevant studies are provided as supplementary material.

### Qualitative analysis

Review findings (*n* = 17) were identified from the primary qualitative research studies using an inductive approach and mapped to six overarching themes. The generation of themes was guided by the ecological model of health behaviour and the social ecology model for health promotion, both of which propose factors beyond the intrapersonal level to influence health behaviour (McLeroy et al., 1988; Stokols, 1992). Each review finding underwent a CERQual confidence assessment (Table 2) and is accompanied by illustrative quotes from individuals across studies to support the finding. Sociodemographic information of participant quotes shared in this review are not provided because of the inconsistency in reporting across studies. Detailed information on the within-category CERQual assessments are provided as supplementary material.

**Table 2.** Summary of the overall confidence rating in each review finding (*n* = 17) using the CERQual approach.

Theme	Review finding	Details	Confidence
Political environment – Facilitates change and shifts perspectives	Awareness	Supported by 52 studies with rich data, minor coherence, and methodological concerns	High
	Facilitates behaviour change	Supported by 60 studies with rich data, minor relevance, coherence, and methodological concerns	High
	Unanticipated positive outcomes	Supported by 39 studies with limited data richness, minor coherence, and methodological concerns	High
Political environment – Restricts freedoms and highlights hypocrisy	Economic concerns	Supported by 20 studies with mostly rich data, and minor methodological concerns	Moderate
	Critiquing the state and its laws	Supported by 53 studies with rich data, minor coherence, and methodological concerns	High
	Loss of freedoms	Supported by 33 studies with mostly rich data, minor coherence, and methodological concerns	High
Sociocultural environment – Group formation	Cultural identity	Supported by 34 studies with rich data, minor relevance, and methodological concerns	Moderate
	Societal attitudes	Supported by 51 studies with rich data, minor relevance, and methodological concerns	High
	Social exclusion	Supported by 33 studies with rich data, moderate relevance concerns, and minor methodological concerns	Moderate
Sociocultural environment – Adapting to the new normal	Adjustment period	Supported by 54 studies with rich data, and minor methodological concerns	High
	Lifestyle disruption	Supported by 46 studies with rich data, and minor methodological concerns	High
	Mirroring others	Supported by 17 studies with limited data richness, moderate relevance concerns, and minor methodological concerns	Low
Sociocultural environment – Social responsibility	Empowerment	Supported by 31 studies with rich data, and minor methodological concerns	Moderate
	Respect and serving society	Supported by 50 studies with rich data, and minor methodological concerns	High
Physical environment – Barriers dictate behaviours	Context-dependent compliance	Supported by 23 studies with limited data richness, minor relevance, and methodological concerns	Low
	Comfort as a driver for behaviour	Supported by 45 studies with rich data, minor relevance, coherence, and methodological concerns	Moderate
	Original behaviour is tied to other practice/setting	Supported by 22 studies with rich data, minor relevance, and methodological concerns	Moderate

### Political environment - facilitates change and shifts perspectives

#### Awareness (*n* = 52)

Knowledge was linked to support for both smoke-free policies and COVID-19 restrictions. Participants across COVID-19 literature sought to educate themselves and most reported complying with guidelines. However, there were challenges associated with mixed messages, unclear rules, public uncertainty surrounding the pandemic, and constantly evolving health guidelines from various levels of government.

“I think that is the scary thing about it you know, where is it all going to end, because people keep demanding an exit strategy, but they can't have a strategy, as they don't know, there is lots of things they don't know.” (Brooke and Clark, 2020)

“Now it’s really hard to know what’s okay to do. I’d like some nice clear information, and clear instruction that wasn’t contradictory. But it’s impossible to get.” (Eraso and Hills, 2021)

Across smoke-free policy literature, some smokers believed smoking harms were exaggerated and risks from negative health outcomes could be avoided through diet and exercise. This was unique to studies where legislation was recently implemented and studies which included older participants who spent most of their lives without anti-smoking messaging and smoke-free spaces (Chief et al., 2016; Hargreaves et al., 2010; Helweg-Larsen et al., 2010; Louka et al., 2006). However, most studies investigating smoke-free policies found that education bolstered societal support for smoke-free spaces.

“[L]ots more people now are aware of the risks and damages that are done…um…advertising per se is a lot more in your face about smoking. [pause] You didn’t have such graphic accounts of what would happen to you 10 years ago, so yeah, I think the part that organizations play in negatively showing smoking has had quite an impact on people's positive attitudes to not smoking.” (Louka et al., 2006)

##### Facilitates behaviour change (*n* = 60)

Implementation of both sets of policies resulted in greater compliance with restrictions and guidelines across most regions. Many participants reported reducing their cigarette consumption, quitting, staying quit, and following COVID-19 guidelines because of factors such as increased self-efficacy, habits, environmental constraints, and changing social norms. During COVID-19, some reported going beyond the recommended guidelines such as wearing gloves, and changing clothes when returning home:

“Now that I can’t smoke in the bars, that is a HUGE help. I smoke a lot less now. I thought that I would have a huge problem with smoke-free bars but I like it a lot; it really needed to be done. It does help a lot.” (Widome et al., 2011)

“...we are careful and religiously obeying the government regulation of physical distancing, self-isolation, and frequent handwashing to avoid health crisis within the shophouse [residence].” (Zwain, 2021)

Smokers who had retained their old habits were from regions where the legislation was newly implemented or areas where the law was not observed (e.g., China, Greece) (Li and Collins, 2017; Louka et al., 2006). Similarly, some rural US and Hispanic communities also experienced a general reluctance to adopt new COVID-19 preventive behaviours (Koon et al., 2021; Moyce et al., 2021).

##### Unanticipated positive outcomes (*n* = 39)

The introduction of smoke-free policies and COVID-19 restrictions led to participants reporting some benefits. Both smokers and non-smokers stated that smoke-free venues were better because they accommodated everyone, and some even cited establishments had made it comfortable for smokers by providing amenities (Hackshaw, 2010; Ritchie et al., 2010b).

“Oh man, I find it great, I find it brilliant. That means you can go home after a night out having pints, and get up then the next day and you're able to put on the same clothes again. So I actually think it’s great.” (Satterlund et al., 2012)

“The pub that I drink in has been fantastic with the smoking ban, they’ve put out a big gas heater…and it’s got a canopy; he has got a gazebo over it. And a couple of folding chairs and what have you, it’s actually quite nice.” (Ritchie et al., 2010b)

Regarding COVID-19 restrictions, some studies found stay-at-home orders led to people spending more time with family, reflecting during idle periods, and developing a stronger faith; some also mentioned they had saved time by not having to commute to work (Ares et al., 2021; Bozdağ, 2021; Sweet et al., 2021; Williams et al., 2020). Many participants, especially older adults, cited technology and embraced learning to use these tools (Fristedt et al., 2021; Kotwal et al., 2021).

“[I]t was also really nice to have a reason not to leave the house and to be able to say we’re just going to stay home and have this quiet time as a family.” (Sweet et al., 2021)

### Political environment - restricts freedoms and highlights hypocrisy

#### Economic concerns (*n* = 20)

Participants across COVID-19 and smoke-free policy studies reported economic concerns for communities and businesses regardless of whether they supported or rejected these rules. Some even admitted these measures were necessary because businesses were driven by profits rather than moral obligations toward customer health:

“There were businesses that would not allow smoking because they care about their customers and didn't want them exposed to smoke. But then they would lose business, where the other bars would say, ‘Smoke, we don't care.’ Now it makes it a more fair because you're not punished for caring about your customers.” (Berg et al., 2011)

“My husband had a [Temporary Workforce Reduction Plan in Spain] and this will directly affect our economic security, and I think this has affected me pretty much.” (Günther-Bel et al., 2020)

##### Critiquing the state and its laws (*n* = 53)

This finding was strongly supported across both smoking and COVID-19 literature. Smokers believed public spaces with open air such as beaches, parks, and bus stops should be exempt from smoke-free legislation. Others even pointed out the hypocrisy of the government to implement regulatory policies while still allowing the sale of cigarettes, and lack of control of other respiratory pollutants.

“I stood at a bus stop one time and all these buses would pull up and you are just inhaling these diesel fumes, and I am leaning on this railing and I light a cigarette and a woman came walking up and she started leaning on the railing beside me. And when she saw me…she gave me a dirty look and moved closer to the exhaust of that bus. Now what is wrong with that whole picture, you know, I mean, oh.” (Betzner et al., 2012)

This had a clear impact on compliance because it reflected that the government was not invested in these health outcomes. As one participant from a study in Greece reported:

“[N]othing applies, it’s just hot air. In essence there's neither law, nor state, nor anything - nor sanctions. As if everything were done for the sake of appearances.” (Benincasa, 2019)

Regarding COVID-19 restrictions, most participants reported observing others disobeying guidelines. Participants were skeptical of the feasibility of enforcement, frustrated with mixed messaging, and critical of leadership:

“I don’t trust this government to fully tell the truth. In fact, given their track record over the last ten years, they lie, underfund vital services and appear not to care about the general population. They care about making money and their rich buddies.” (Enria et al., 2021)

“I have seen loads of people outside, and I wonder how people will enforce that…” (Williams et al., 2020)

“I don't know why it took so long [to recommend mask use]. It just doesn't even seem logical when they were saying wearing a mask doesn't stop the spread or slow the spread. I don't know what they were thinking.” (Lee et al., 2021)

##### Loss of freedoms (*n* = 33)

Studies investigating COVID-19 and smoke-free policies both commented that individuals perceived these rules as an infringement on personal rights and compared themselves to being prisoners in their own communities. During the pandemic, participants also tied it to social isolation.

“There’s just more rules. It is a problem ‘cause people go out to enjoy themselves and to be free and you’re being restricted, people aren’t gonna want to go out.” (Wakefield et al., 2009)

“Sometimes, I think they are exaggerating and that they could ease up on their enthusiasm to limit personal freedom.” (Kamin et al., 2021)

“[W]e are not great at being dictated to, or doing things that are for our benefit...and it’s going to get worse from a civil unrest point of view. I fear we are sleepwalking into a police state.” (Williams et al., 2021)

Smokers believed smoke-free policies should be dictated by the venue rather than government.

“I think it should be up to the business to allow it or not to allow it. And not the government telling us, you shouldn’t do this, because again with the slippery slope. Where does it end?” (Crosbie et al., 2020)

### Sociocultural environment - group formation

#### Cultural identity (*n* = 34)

Across smoking and COVID-19 literature, differences were observed in societal norms across cultures and religions regarding preventive health behaviours. In Greece, China, Denmark, Irish-American and Indigenous communities within the US, smoking was generally more acceptable (Benincasa, 2019; Chief et al., 2016; Helweg-Larsen et al., 2010; Louka et al., 2006; Satterlund et al., 2009). In contrast, the US, Canada, and the UK experienced cultural shifts due to awareness of smoking harms and effective legislation towards anti-smoking (Bell et al., 2010; Betzner et al., 2012; Hargreaves and Highet, 2009; Li and Collins, 2017).

“[In France] the people themselves can support that. In Greece, the fact that something is forbidden doesn’t mean anything. We're a little…How shall I put this…The fact that a law exists doesn’t mean that it is observed. And that's not only about smoking, it's the same in many other things.” (Benincasa, 2019)

In some cultures, smoking was only accepted among men. This was observed by Bangladeshi, Chinese, Indian, and Somali immigrants, and international students (Highet et al., 2011; Li and Collins, 2017; Lock et al., 2010; Tan, 2013). Among some groups, COVID-19 restrictions were associated with pre-existing gender norms and traditions (Alqahtani et al., 2021; Shelus et al., 2020), but this finding was not strongly supported across studies.

“It’s more a taboo…and that’s one of the things that’s…relevant in Somalia. Yes, woman smokers are looked down on…you know, more than men.” (Lock et al., 2010)

“…staying at home and covering the face is not for men in our culture…” (Alqahtani et al., 2021)

Religious and cultural norms were salient after the implementation of COVID-19 restrictions. Some studies out of the US revealed participants put their faith in God or felt the pandemic was in God’s hands (Koon et al., 2021; Moyce et al., 2021; Shelus et al., 2020). Although participants expressed difficulty following government mandates during the pandemic, many commented on preventive behaviours positively by tying it to their religious, ethnic, or cultural identity.

“…from a Muslim perspective we are very sociable as a community or communities, whether you’re sort of Arab, Asian, African you tend to have backgrounds of living if not with family having a lot of involvement with your family even day to day interactions…so I think there’s lots of that physical contact is very much part of it hugging and shaking hands and so I think there’s a combination of things that probably makes us more at risk.” (Hassan et al., 2021)

“The first and most important command in the Torah is ‘take good care of your life’. Even when the synagogues closes, your life comes first.” (Vanhamel et al., 2021)

“I think the Japanese don’t want to bother other people in the society by being infected by the COVID-19.” (He and Traphagan, 2021)

“[W]hen I saw a few people wearing masks…I was like, yes, that person’s Asian. It’s just Asian people wear masks…when you’re sick to be polite.” (Zhang et al., 2022)

##### Societal attitudes (*n* = 51)

There was a shift in the social acceptability of preventive health behaviours across both smoke-free policy and COVID-19 studies. This was due to legislation as well as messaging and education, but the result was a change in societal norms.

“If you look broadly at the population you see that [smoking] is socially unacceptable. It is just un-trendy in many circumstances and in many people’s eyes…It is just not cool any longer and if you smoke and people do not really know you then they might put you in a group with people who do not have control over the situation, who are not clever or smart, or are not so well educated.” (Helweg-Larsen et al., 2010)

“If I don’t wear a mask, I’ll be looked at coldly.” (Takashima et al., 2020)

Negative sentiments were expressed across all COVID-19 studies, but studies differed in the acceptability of norms. The literature indicated norms were changing to support new preventive behaviours (He and Traphagan, 2021; Kim et al., 2021; Koon et al., 2021; Moss and Sandbakken, 2021), but there were rare instances where some communities rejected them (Mollborn et al., 2021).

“Entender que nadie se salva solo. Si nos cuidamos y prevenimos se evitan más muertes.” [Understand that no one is saved alone. If we take care of ourselves and we prevent, more deaths are avoided.] (Cecilia Johnson et al., 2020)

“Even those who joked more than me, who were very much like, ‘oh my god, it’s just blah blah blah again about virus and stuff like that, it’s just hysteria from the entire society’…They are now very much siding with the measures and understand that it has to be this way.” (Moss and Sandbakken, 2021)

“You feel the pressure…If you don’t go out, it’s like you’re dumb or you live in fear or you’re letting the government control your rights.” (Shelus et al., 2020)

“A lot of people not wearing masks. I don’t see them keeping their social distance….My perception is a lot of people don’t think it’s very serious…” (Mollborn et al., 2021)

##### Social exclusion (*n* = 33)

This finding was supported by both smoke-free policy and COVID-19 studies. Virtually all smokers experienced feelings of stigma, embarrassment, and social exclusion due to changing social norms.

“I think it; it's sort of, sort of making, making third class citizens out of the smokers, to actually have to put them on, on show. It's almost like being put in the stocks and pilloried, you know, by the rest.” (Hargreaves and Highet, 2009)

“Well that's all changed now, hasn't it? And that's changing the stigma attached to smoking - the more it's shifted - so you're a social outcast if you smoke cigarettes.” (Parnell et al., 2019)

The pandemic and its restrictions resulted in exclusion of certain groups including Jewish people, Asians living in Western societies, and older adults.

“It is often like that, when a problem emerges in the world, that the Jews are singled out…there was an article in the beginning that stated that Orthodox Jews were not adhering to the rules of social distancing and that 500 Jews will die from corona...[it] can be used as anti-Semitism.” (Vanhamel et al., 2021)

Stigma was often present in the form of a perceived increased community vigilance regarding following restrictions and guidelines.

“I was thinking that neighbors are watching and wondering why I’m going to do the shopping.” (Lohiniva et al., 2021)

“People are starting to judge others if they don’t wear [a face mask] so it’s better to just wear it.” (DeJonckheere et al., 2021)

“Like yesterday, when we were standing out on the sidewalk and talking with a friend, then I was conscious of it when people walked past. A woman who walked past us looked at us very intently.” (Moss and Sandbakken, 2021)

### Sociocultural environment - adapting to the new normal

#### Adjustment period (*n* = 54)

This finding was supported across smoking-related and COVID-19 studies. Smokers indicated they were adjusting to the legislation just as other smokers and establishments were as well.

“A lot of places are just adjusting. Places are making an [sic] outdoor smoking areas. You can go out and it’s actually warm. People will adjust, and it will be fine again.” (Berg et al., 2011)

During the pandemic, individuals developed resilience through the hardships and restrictions. Participants experienced loneliness but adjusted by organizing at-home family activities, arranging phone calls with friends, learning to use technology, participating in online exercise classes, and gardening (Brooke and Clark, 2020; Chen et al., 2021; Fristedt et al., 2021). At times, the restrictions and preventive behaviours expressed by participants seemed to show signs of habit formation.

“It used to be difficult and bothersome [to wear a mask] and I often forgot to do it...Now I look for the mask first thing in the morning and don’t even realize that I am wearing a mask all day.” (Kim et al., 2021)

##### Lifestyle disruption (*n* = 46)

Participants across both sets of studies cited disruption to their opportunities for socialising. Smokers reported having to interrupt conversations to go out for a cigarette and changing their routines to smoke more at home instead of going out. The public during the COVID-19 pandemic experienced a greater number of restrictions, and changes to their routines and travel plans.

“If you’re sitting having a conversation and you just get up and go and have a cigarette and come back down, it’s not very nice. You seem to lose track of what’s happening in the club if you’re outside all the time.” (Ritchie et al., 2010b)

“Not being able to go to stores and feel merchandise; like fabrics of clothing, etc. Not being able to enjoy a cup of coffee out at café.” (Heid et al., 2021)

“I changed my life. I can’t see my children and grandsons…they live in Tokyo. Tokyo has many COVID-19 cases…I told them don’t come back home until COVID-19 is done.” (He and Traphagan, 2021)

##### Mirroring others (*n* = 17)

This review finding expands on how social cues influence individuals to follow behaviours. Particularly, it was linked to the social context of smoking and cues to action in COVID-19 studies. Smokers and ex-smokers alike acknowledged that others smoking in a bar or club would impact their self-efficacy to not smoke. Furthermore, participants reported people within their social circle had a strong influence in their decision to smoke, both positively and negatively.

“Yeah, I’ve quit but like when I stay around smokers...I’ve still got that feeling that I want one. And when I’m drinking I have one or two…Yes, if like my friends will tell to go on ‘Do you want one?’…like, so I go ‘Okay’ [laughs], you know…if I’m drinking. Just the weekend, you can say one or two.” (Hargreaves et al., 2010)

COVID-19 preventive behaviours such as physical distancing and wearing face masks were followed or not followed based on cues from others (Burton et al., 2022; Schönweitz et al., 2022):

“[Canada] is a vast and sparsely populated country. Unlike China, which requires people to stay away from supermarkets and to wear masks, Canadians here rarely wear masks, so I don’t wear masks either.” (Wang et al., 2021)

### Sociocultural environment - social responsibility

#### Empowerment (*n* = 31)

Individuals were more likely to discuss risks of unsafe behaviours with their friends and family in both COVID-19 and smoking contexts. People imposed stronger rules on their family members during the COVID-19 pandemic:

“Keep your friends and relatives accountable during this pandemic. I have had to have a very direct conversation with my friend because she is taking unnecessary dangerous risks which will impact the course of this virus.” (Chen et al., 2021)

“[O]ur children are more worried than we are, saying you have to stay indoors. You can’t go shopping. You can’t go anywhere.” (Fristedt et al., 2021)

On the other hand, interactions with strangers were more hostile in both COVID-19 and smoke-free policy studies. This was observed in Western societies which had normalized confronting strangers or making remarks about others’ noncompliance post-legislation (Bell et al., 2010; Hargreaves et al., 2010; Louka et al., 2006). However, these actions were not culturally acceptable in other countries.

“At bus stops, I even told a few people, ‘Uh, excuse me, could you please stop smoking here?’ They told me…I think they gave me dirty looks to say, ‘Why don't you die?’ [laughs]” (González-Salgado et al., 2020)

“Even if you see someone smoking in smoke-free spaces [in China], you do not have the rights (sic) to stop him.” (Li and Collins, 2017)

##### Respect & serving society (*n* = 50)

Common courtesy and being considerate were important to most participants. Smokers reported blowing smoke away from others, moving farther away when non-smokers were in the vicinity, and not wanting to interrupt the flow of conversation inside a venue by leaving for a cigarette.

“I try to be considerate, you know, and hold my cigarette away or try and blow it in a different direction to the person I’m sitting with.” (Hargreaves and Highet, 2009)

For non-smokers and individuals during the COVID-19 pandemic, following the rules was often associated with being responsible, respectful, and united as a community. Those not following rules were perceived as being careless and irresponsible.

“…when we are out in public, we are wearing masks…more out of respect to the community than out of concern for our safety.” (Mollborn et al., 2021)

“I think it’s just kind of a respect thing. So, I respect you, you respect me. We all wear a mask…keep each other safe.” (Lee et al., 2021)

“It's a respect thing. It's the fact of respecting not to smoke inside and the kids are aware of that. They are raised that way around here, but to a certain extent. Just being respectful that's all…” (Chief et al., 2016)

Additionally, serving the community was influenced by concern for vulnerable groups, being role models for children, and not wanting local businesses and staff to be reprimanded for customers breaking rules (Chen et al., 2021; Moss and Sandbakken, 2021; Nilsson et al., 2021; Rhodes et al., 2021; Ritchie et al., 2010b).

### Physical environment – barriers dictate behaviours

#### Context-dependent compliance (*n* = 23)

Noncompliance was often related to the ambiguity of smoke-free policies and COVID-19 restrictions, greater perceived risk in some public areas, and inability to practice preventive behaviours. Participants observed or reported breaking rules at bus stops, railway stations, snooker halls, supermarkets, public parks, and culturally diverse cafés:

“…I have had bus drivers not let me on the bus [b]ecause I was smoking at a bus stop. Open, not even covered, like. Open, nobody around me. There isn’t like a three-year-old child next to me, and I’m not breathing smoke in their face or anything, and by myself smoking and they like won’t let me on. They haven’t let me on the bus.” (Bell et al., 2010)

Many participants cited it was not possible to follow guidelines because of how public spaces were designed (Benham et al., 2021; Hassan et al., 2021; Moss and Sandbakken, 2021):

“I try to stay 2 m away from everyone however this is not always possible in a small shop and most customers do not care about social distancing.” (Leather et al., 2022)

##### Comfort as a driver for behaviour (*n* = 45)

Comfort, enjoyment, and convenience were drivers for behaviour change within the smoke-free and COVID-19 literature. Smokers generally reported it was inconvenient to exit a venue to smoke cigarettes, which often resulted in reducing the frequency of use and visits to these establishments:

“[As a result of the smoke-free legislation] you end up probably smoking less. Because you physically have to go and brave the wet weather or the cold [laughs] and it’s actually, you, if you, if I was smoking at my desk I’d smoke a lot, lot more.” (Juszczyk and Gillison, 2019)

“[I]t is just a major inconvenience. It doesn't really affect you…[it] is a big pain in the neck…but as far as really motivating me to quit, I don’t think it has really done that.” (Betzner et al., 2012)

Non-smokers and some smokers stated they were more comfortable in restaurants, cafés, and bars after smoke-free legislation was implemented:

“I thought it was a really good idea…because it was just very unpleasant to be in, even though I was a smoker, I didn't like sitting in smoky rooms because of your hair, your clothes, watering eyes, all of that kind of stuff, I didn't like sitting in rooms that were full of smoke.” (Hackshaw, 2010)

Communities providing smoking sections and shelters were reported as being uncomfortable due to poor ventilation, lack of protection from weather, or embarrassing to be in:

“The bad weather, if it's likes of raining and what have you because I mean they've got this stupid wee bus shelter down there and the way it's facing is you're open to the weather coming from the West and it comes from the West anyway." (Ritchie et al., 2010a)

Weaker evidence was found from COVID-19 studies to support this domain. However, these studies revealed individuals were re-evaluating the safety of public spaces. This resulted in many opting to spend more time at home and following stay-at-home orders.

“There is no safe place. I cannot go to even a convenience store or a market.” (Takashima et al., 2020)

“Staying home during the COVID-19 pandemic is like hiding from [an] enemy because when I go out, I feel I have an enemy that can harm me any time.” (Bozdağ, 2021)

##### Original behaviour tied to other practice or setting (*n* = 22)

This finding was supported across both smoke-free studies and COVID-19 literature. Smoking was associated with other behaviours (e.g., drinking coffee, alcoholic behaviours, socializing) and settings (e.g., restaurants, bingo halls, cafes, nightclubs, bars and pubs):

“What bothers me most? Yes, not being able to go into a café and have a cigarette. Is the most, for me, because I always did that, you know, and that [was] three times a week.” (Hargreaves and Highet, 2009)

“In some bars you were allowed to smoke inside and in others you weren't. So then I went to bars where it was allowed. And at a certain point, it wasn't allowed there anymore either. And then I stopped going. After that, I tried to go there twice, but I missed the pleasurable atmosphere and a tasty cigarette with a drink.” (Van der Heiden et al., 2013)

“The more beer I have, the more cigarettes I seem to have. And they just seem to go down so well with the drink.” (Wakefield et al., 2009)

COVID-19 preventive behaviours were similar, where some aspects of socializing and normalcy were affected:

“Now everything is a controlled environment…everything is controlled and clear, and in a controlled environment, there is no place for surprises that make up your day. Those random encounters - I do not know, I miss that. A controlled environment is very predictable, it gets boring.” (Kamin et al., 2021)

“I wear the mask to save a social situation...and I think I care a lot that we kind of maintain certain social contexts as long as we can. And I would like to contribute to making that possible.” (Schönweitz et al., 2022)

## Discussion

### Overcoming reporting and knowledge gaps

A large body of qualitative literature was reviewed and synthesized to explore relationships and key environmental factors of preventive health behaviours related to smoke-free policies and COVID-19 restrictions. This was supplemented with established qualitative knowledge synthesis methods (Thomas and Harden, 2008). Studies in this review generally embedded their investigation of environmental factors with interpersonal and individual factors explaining behaviour. Additionally, most studies did not have a policy focus and instead discussed restrictions in general. Therefore, we identified a need for qualitative research in this area to narrow the focus of the investigation to one or more environmental factors and specify setting(s) of interest. Most relevant articles were published out of the US, UK, and Canada, which leaned toward Western perspectives and environments. In fact, some studies were multinational (Brooke and Clark, 2020; Helweg-Larsen et al., 2010; Louka et al., 2006) or focused on migrants and visible minorities (Highet et al., 2011; Li and Collins, 2017; Vijayaraghavan et al., 2018), which revealed regional and cultural differences in norms and perceptions of these policies and preventive behaviours. We advise caution as these review findings may only be applicable to Western-centred investigations and settings. Other qualitative research have explored COVID-19 challenges among at-risk groups (García-Martín et al., 2021; Mackworth-Young et al., 2021; Rhodes et al., 2021; Sun et al., 2020; Takashima et al., 2020; Wang et al., 2021), yet we found a gap in investigations among people of low socioeconomic status.

Studies identified in this review met most quality assessment criteria. However, gaps in reporting were found to be mostly in the qualitative approach used and describing analyses in sufficient detail. Outlining how study findings and themes were generated are critical contributors to study reproducibility and transparency (Pope et al., 2007). Future qualitative research should report the following, if applicable: qualitative design, guiding theories, research paradigm, rationale for data analysis, and process by which themes or main findings were generated. Furthermore, several studies lacked information on ethical considerations and researcher reflexivity. Researchers conducting primary qualitative research in this area are strongly urged to follow reporting guidelines (O’Brien et al., 2014; Tong et al., 2007), which call on authors to report key details such as ethics review board approval, participant consent process, researcher characteristics, and the researcher-participant relationship.

### Research implications

Overall, three overarching findings were identified: (a) the political environment facilitates behaviour change, shifts perspectives, and restricts freedoms; (b) the sociocultural environment promotes group formation, results in the adaptation to a new normal, and highlights social responsibility; and (c) the physical environment affected comfort and likelihood to perform a health behaviour.

It was evident the political environment determined the level of compliance of preventive health behaviours. Restrictive legislation prompted many within the populations studied to comply with public health’s intentions (Centers for Disease Control and Prevention, 2020; Frazer et al., 2016; Lee et al., 2011). However, initial opposition and negative consequences arose when such policies were introduced, such as skepticism, economic concerns, and perceived threat to freedoms. This may have been the result of insufficient public knowledge or poor attitudes relating to harms associated with the unsafe behaviour(s) prior to, during, and after implementation of these policies. A multinational study on smoke-free legislation found awareness of smoking harms was associated with support for smoking bans (Mons et al., 2012). Furthermore, studies have demonstrated that stronger smoke-free regulations were associated with improved attitudes and support for smoke-free policies (Hyland et al., 2009; Mons et al., 2012), especially over time (Hyland et al., 2009; Thomson et al., 2016), suggesting acceptance and support increase once the public experiences benefits from community-wide measures. Going forward, public health practitioners and policymakers can consider the following to minimize public opposition, bolster support, and improve behaviour uptake: outlining the extent of bans and harms of unsafe behaviours via public awareness campaigns; using evidence to explain the value of prevention (Brownson et al., 2009); highlighting benefits of restrictions post-implementation (e.g., deaths prevented, potential outbreaks avoided); and opting for stronger restrictions that encompass all community settings (vs. partial restrictions that are context-specific) during crises despite initial debate. If widespread bans are not feasible, restrictions can be widened to other settings over time assuming policy evaluations demonstrate the effectiveness of partial restrictions (Brownson et al., 2009).

The political environment shaped and was shaped by the sociocultural environment in many ways. In other words, the enforcement of certain restrictions labelled a behaviour as inappropriate in the community, which facilitated changes in long-term public perceptions and establishing new social norms. Smoking became denormalized and there were cultural norms, societal pressures, and stigma towards smokers and certain groups (e.g., immigrant women) to not smoke. Regarding stigma, there was some evidence from our review findings to support the environmental influence on COVID-19 preventive health behaviours. For example, people were vigilant of others’ behaviours in public, some immigrant men associated staying at home and wearing masks as feminine, and individuals who were previously infected with COVID-19 or who were older adults felt excluded from society. Studies have suggested gender norms mediate smoking-related intentions (Chinwong et al., 2018; Morrow et al., 2002) and intentions to wear masks (Bhasin et al., 2020; Capraro et al., 2020) which affects the (de)normalization of these health behaviours. This suggests health behaviours practiced in public spaces may be assigned masculine or feminine traits depending on the region and culture. However, these norms seem to be diminished or eliminated in regions where regulatory policies to support healthy behaviours are already in place (Capraro et al., 2020). Stigma was closely linked to group formation (e.g., smokers and non-smokers). Groups have also been formed on health behaviour sentiments during the COVID-era, with the resurfacing of anti-vaccine sentiments (Burki, 2020; Center for Countering Digital Hate, 2020) and pro- and anti-masker groups (Bhasin et al., 2020; Capraro et al., 2020; He et al., 2021). More research is needed on religious norms and normalization of physical distancing and wearing masks as these topics were underrepresented in the included studies.

The sociocultural environment also revealed concern for vulnerable groups and the greater community as important drivers of preventive health behaviours and support for community restrictions. Quantitative studies also showed similar findings (Thomson et al., 2016; Yang et al., 2013), where support for regulatory health policies was greater in areas where vulnerable populations were at risk, such as schoolgrounds (Thomson et al., 2016). Being considerate, respectful, and cognizant of social norms in public have been demonstrated in the smoking and COVID-19 literature (Leather et al., 2022; Poland, 2000; Schönweitz et al., 2022), suggesting a collective identity, in this case achieving a common end goal of smoke-free spaces and returning to normal with COVID-19. For instance, one study found intentions to practice COVID-19 preventive behaviours improved when health messaging contained words such as family and community (Capraro et al., 2020). Thus, social acceptability of favourable behaviours can be prioritized using moral messaging by outlining risks to the community.

Lastly, the physical environment influenced preventive health behaviours. Environmental constraints prevented individuals from adhering to restrictions despite their intentions. This indicates broader changes may be needed to redesign public spaces and establishments to enable healthy behaviours and improve self-efficacy, which is a strong determinant of health behaviours (Hamilton et al., 2020; Lin et al., 2020; Macy et al., 2012). Occasionally, a lack of awareness also resulted in non-compliance; this can be reduced through federal support for municipalities to place relevant signage in regulated areas (Mark et al., 2014). Previously established habits in outdoor areas and indoor venues were reportedly difficult to eliminate, and some quantitative research show habits predict intentions to follow public guidelines (Hagger et al., 2020). Based on previous assumptions and evidence, there may be a (de)normalization process where preventive behaviours eventually reach favourable levels of acceptance and support after implementation of regulations (Hyland et al., 2009; Schönweitz et al., 2022; Thomson et al., 2016).

### Topic-specific differences

COVID-19 restrictions and smoke-free policies differ in many ways and the nuances cannot be understated. Measures during the COVID-19 pandemic were newly enacted to limit community transmission of the virus during a global crisis (Williams et al., 2021), whereas smoke-free policies are an established measure to deter smokers from smoking in shared community spaces and protect others from second-hand smoke (Betzner et al., 2012). Although all themes were supported by evidence from both topics, the physical environment category had insufficient evidence from COVID-19 studies to strongly support some review findings. These findings may be unique to smoke-free compliance. Nicotine dependence is an important factor in cessation behaviour (Transdisciplinary Tobacco Use Research Center (TTURC) Tobacco Dependence et al., 2007) and an increase in dependence is associated with lower perceived behavioural control (Macy et al., 2012). The enforcement of smoke-free spaces may result in smokers experiencing discomfort caused by nicotine dependence, and therefore decreased perceived control to comply in these venues. Furthermore, cigarette use can be reinforced with other substances such as coffee or alcohol (Jiang and Ling, 2011; Marshall et al., 1980), and beverages available at these establishments may create a co-dependence. Additionally, mirroring others was not found to be mentioned broadly across the COVID-19 studies in this review. Despite these differences, a cross-comparison and synthesis of COVID-19 with smoke-free policies provided a novel understanding on how environmental factors shape individual health behaviours in community settings.

### Considerations for quantitative research

Future studies using social cognition models may benefit from the inclusion of some variables identified in this review. Key environmental factors include living in a region where regulatory health policies are present; awareness of the extent of the regulatory health policies; cultural norms that attach a gender or age group to a health behaviour; societal values that attach morality to the desired health behaviour (i.e., moral norm); and the presence of environmental constraints. Some studies have successfully used these variables to explain smoking- and COVID-19-related preventive behaviours (Bogg and Milad, 2020; Clark et al., 2020; Hagger et al., 2020; Macy et al., 2012). Modifying social cognition models to include these factors can: strengthen health behaviour research; explain the environmental impacts on behavioural attitudes and intentions; and provide public health researchers with improved recommendations for behaviour change interventions that support proposed and existing regulatory health policies.

### Limitations

A few limitations were identified. Poor indexing of qualitative research by databases may have resulted in the omission of relevant studies in this review (Shaw et al., 2004). However, efforts were made to follow previous recommendations to make the search strategy over-inclusive to draw out relevant articles which would otherwise have been missed (Shaw et al., 2004). In addition to this, we searched literature across multiple databases, performed searches for grey literature, and hand searched reference lists of select relevant articles. We also acknowledge that COVID-19 was still an emerging area of research at the time of writing this manuscript. However, this was mitigated by conducting an updated database search 2 years after the initial search which resulted in a high degree of coverage of COVID-19 and smoking literature. There was also a possibility that policy-specific themes were drowned out by the inclusion of multiple areas of research in this review. However, we believe the inclusion of smoke-free policies in combination with COVID-19 restrictions allowed for a larger sample of studies and data to saturate potential findings from our thematic synthesis. The resulting findings and themes can also be applied to a broader range of public health issues than if the review had focused on one topic. Lastly, it may be argued CERQual assessments invite subjectivity into the review process. This was reduced by having a second reviewer validate findings. Although this did not eliminate subjectivity, the aim of CERQual is not to make an objective assessment of confidence; rather it is completed to identify potential threats to the evidence and allow for transparency of judgements of evidence (Lewin, Bohren, et al., 2018).

## Conclusion

This systematic review used structured and transparent methods to synthesize data from primary qualitative research studies exploring the environmental determinants of chronic and infectious disease preventive behaviours among the public using regulatory smoke-free policies and COVID-19 restrictions as case studies. The three main review findings were: (a) the political environment facilitates behaviour change, shifts perspectives, and restricts freedoms; (b) the sociocultural environment promotes group formation, results in the adaptation to a new normal, and highlights social responsibility; and (c) the physical environment influences an individual’s comfort and likelihood to perform health behaviours. Social cognition models provide a means to investigate health behaviours and these frameworks can incorporate environmental variables identified in this review to better explain attitudes and behaviours. Future primary research should explore the (de)normalization phenomenon of health behaviours and the rate at which they are incorporated into communities. These results can enhance our understanding of current community preventive health behaviours under the direct influence of environmental factors and provide insights to improve the implementation of evidence-informed regulatory health policies.

## Supplemental Material

Supplemental Material - Environmental determinants of infectious and chronic disease prevention behaviours: A systematic review and thematic synthesis of qualitative research Click here for additional data file. Supplemental Material for Environmental determinants of infectious and chronic disease prevention behaviours: A systematic review and thematic synthesis of qualitative research by Abhinand Thaivalappil, Anit Bhattacharyya, Ian Young, Sydney Gosselin, David L Pearl and Andrew Papadopoulos in Health Psychology Open

Supplemental Material - Environmental determinants of infectious and chronic disease prevention behaviours: A systematic review and thematic synthesis of qualitative research Click here for additional data file. Supplemental Material for Environmental determinants of infectious and chronic disease prevention behaviours: A systematic review and thematic synthesis of qualitative research by Abhinand Thaivalappil, Anit Bhattacharyya, Ian Young, Sydney Gosselin, David L Pearl and Andrew Papadopoulos in Health Psychology Open

Supplemental Material - Environmental determinants of infectious and chronic disease prevention behaviours: A systematic review and thematic synthesis of qualitative research Click here for additional data file. Supplemental Material for Environmental determinants of infectious and chronic disease prevention behaviours: A systematic review and thematic synthesis of qualitative research by Abhinand Thaivalappil, Anit Bhattacharyya, Ian Young, Sydney Gosselin, David L Pearl and Andrew Papadopoulos in Health Psychology Open

Supplemental Material - Environmental determinants of infectious and chronic disease prevention behaviours: A systematic review and thematic synthesis of qualitative research Click here for additional data file. Supplemental Material for Environmental determinants of infectious and chronic disease prevention behaviours: A systematic review and thematic synthesis of qualitative research by Abhinand Thaivalappil, Anit Bhattacharyya, Ian Young, Sydney Gosselin, David L Pearl and Andrew Papadopoulos in Health Psychology Open
