# Supplementary material for: Environmental determinants of infectious and chronic disease prevention behaviours: A systematic review and thematic synthesis of qualitative research
Source: Health Psychol Open. 2023 May 25;10(1):20551029231179157. doi: 10.1177/20551029231179157 (PMC10226319; doi:10.1177/20551029231179157)
Supplement: Supplemental Material - Environmental determinants of infectious and chronic disease prevention behaviours: A systematic review and thematic synthesis of qualitative research [file sj-pdf-1-hpo-10.1177_20551029231179157.pdf]

Protocol: Environmental determinants of infectious and chronic disease prevention behaviours: A systematic review and thematic synthesis of qualitative research

Review Background and Rationale:

Health behaviour is an activity performed by an individual who perceives an action to be healthy with the primary purpose of preventing disease or detecting it at an asymptomatic stage (Conner & Norman, 2005). The study of health behaviours often include social cognition models which broadly state that an individual's perceptions of their social environment help predict their behaviour (Conner & Norman, 2005). These theories (e.g., Social Cognitive Theory, Health Belief Model, Theory of Planned Behaviour) have been widely used to understand health behaviours and in the development of interventions to influence the prevalence of certain behaviours, thereby improving population health (Conner & Norman, 2005).Unlike other theories of behaviour change, the social ecological model proposes that interpersonal, organizational, community, cultural, and public policy factors influence individual health outcomes with each subsequent factor influencing the ones that precede it (Centers for Disease Control and Prevention, 2020). This was the first framework of its kind to suggest higher-level societal factors (i.e., culture, public policy) exerted pressures on lower levels (i.e., interpersonal, individual) to shape overall behaviour. Due to the complex relationships between factors, the model only presents levels from top-to-bottom (Bronfenbrenner, 1979; Lumen Learning, n.d.).

The impacts of societal factors were pronounced in the adoption and implementation of smoke-free policies (Barnoya & Navas-acien, 2013; Government of Canada, 2015; World Health Organization, n.d.). More recently, impacts were observed in the execution of novel behaviours which emerged during the coronavirus disease 2019 (COVID-19) pandemic, such as wearing face coverings and practicing physical distancing to reduce viral transmission (Beck & Hensher, 2020; Biddlestone et al., 2020). In both cases, some evidence suggests cultural factors shape general attitudes and the level of support for policies and behaviours (Lazuras et al., 2009; Omura et al., 2020). The result was an increased compliance of desired health behaviours and prevention of unsafe behaviours while bypassing the typical route of modifying individual attitudes and beliefs (Barnoya & Navas-acien, 2013; LaRochelle-Côté & Uppal, 2020). However, the influence of cultural norms and enforced health policies on resulting individual attitudes and beliefs is not well understood. Furthermore, it is also unclear which direction trust and support within government moves when such policies are introduced. Therefore, a need exists to understand the impacts of societal factors (e.g., culture, health policy) on individual attitudes and beliefs related to infectious and chronic disease prevention behaviours within the general public.

We propose a systematic review and thematic synthesis using standardized methods to identify, characterize, and synthesize all available qualitative studies examining societal determinants on individual behavioural components (e.g., attitudes, beliefs) using case study behaviours associated with smoke-free policies and COVID-19 restrictions. The results from this review will provide two overarching benefits. First, it will enhance the understanding of health behaviours which can be used to extend existing theories to comprehensively explain health behaviours. Second, study findings can provide clues to the impact policies and cultural norms exert on individual attitudes, beliefs, intentions, and behaviours. This can allow public health practitioners to assess which beliefs and attitudes need to be targeted within certain cultures or during implementation of health policies to maintain public health trust, bolster public support for evidence-backed health policies, and maximize compliance of desired behaviours across communities.

Methodology:

Review approach, question, and eligibility criteria:

This research will be conducted using standard systematic review methodology, which uses structured, transparent, and robust procedures to identify and assess all available evidence on a topic (Higgins & Thomas, 2019). Thematic synthesis methodology will also be used to synthesize qualitative evidence, which involves line-by-line coding, development of descriptive themes, and generation of analytical themes to create new explanations and concepts (Thomas & Harden, 2008). The review question is: *"What are the cultural, societal, and regulatory policy determinants on individuals' infectious and chronic disease prevention attitudes, beliefs, and behaviours?"* The inclusion criteria are as follows:

- Topic = An identified list of public health-backed infectious and chronic disease prevention health behaviours influenced by regulatory policies and culture are the topic of interest. This includes smoke-free policies and COVID-19 restrictions (social distancing, stay-at-home orders, use of face coverings). Laws in place concerned with public safety (vs. public health) will be excluded. These include impaired driving, distracted driving, seat belts, and public intoxication which exist to reduce immediate mortality or injury.

- Population = The general public in a community setting. Also include studies investigating public opinion on policies and societal norms in bars, pubs, and restaurants. Exclude studies investigating: residents in a private setting (e.g., home, multiunit housing, vehicle); workers in an organizational context (e.g., healthcare staff, restaurant managers); institutionalized individuals (e.g., patients, prisoners); and studies interviewing adolescents and children under 18 years old.

- Exposure = Research on societal factors (e.g., regulatory policies and cultural norms) related to smoke-free practices; social distancing, stay-at-home orders, hand hygiene, and mask use during the COVID-19 pandemic.

- Outcome = Research on attitudes, beliefs, perceptions, intentions, and behaviours of chronic and infectious disease prevention behaviours. Secondary outcomes will also include trust and support in governing bodies as they relate to policy implementation. Exclude studies which focus on secondary health outcomes only (e.g., mental health issues, dietary behaviours).

- Study = All primary qualitative and mixed methods research designs will be considered for inclusion from countries that the United Nations categorizes as Very High Human Development (United Nations Development Programme, 2019). Eligible sources of evidence will include journal articles as well as any other research documents (e.g., research reports, dissertations and theses, and conference proceedings) published in English, French, or Spanish.

Search strategy:

A comprehensive search strategy will be developed using a combination of pre-tested search terms implemented in the following bibliographic databases: PubMed, Web of Science, PsycINFO, Sociological Abstracts, and ProQuest Dissertations and Theses. The proposed search algorithm is shown below:

Category	Terms
Topic	"COVID-19" OR "SARS-CoV-2" OR "nCoV" OR "smoke-free" OR "tobacco-free"
Population	resident* OR "older people" OR adults OR smokers OR communit* OR population* OR migrant* OR customer* OR Indigenous OR "college student*" OR "university student*"
Exposure	enforc* OR restrict* OR policy OR policies OR bylaw OR "by-law" OR

	constitution* OR ban OR bans OR bann* OR regulat* OR legislation* OR mandat* OR illegal OR law OR laws OR "social distancing" OR "physical distancing" OR "wearing mask*" OR "face mask*" OR "face covering*" OR "stay-at-home" OR "shelter-in-place" OR lockdown OR quarantine OR "public health order*" OR "government order*" OR "government-issued" OR "government response*" OR cultur* OR customs OR tradition* OR religio* OR "societal norm*" OR "social norm*" OR "societal context"
Outcome	knowledge OR awareness OR attitud* OR opinion* OR belief* OR perceive* OR perception* OR motivat* OR concern* OR challeng* OR trust OR distrust* OR support OR barrier* OR facilitator* OR intention* OR behavior* OR behaviour* OR compliance OR adhere*
Study Type	qualitative OR "focus groups" OR "thematic analysis" OR "semi-structured" OR "open-ended questions" OR "key informant" OR ethnograph* OR "in-depth" OR indepth OR "face-to-face" OR "mixed methods"

Categories will be combined using the AND operator. This combination of terms was pre-tested in PubMed. **Number of hits in PubMed (29 October 2020) = 363.**

A complementary search for grey literature documents (e.g., conference proceedings and research reports) will be conducted in Google and Google Scholar. A search verification strategy will also be employed to ensure that no relevant articles are missed, which will include hand-searching the reference lists of a selection of relevant articles.

Relevance screening and confirmation:

The titles and abstracts of citations identified during the search will be assessed for their relevance using a structured screening form (Appendix A). Full articles of relevant references will be obtained, they will be confirmed for relevance, and key characteristics will be extracted using another structured form (Appendix B). This form will capture study characteristics such as: publication type and year; study methods and context (e.g., design, data collection methods); and details on the target populations (e.g., socio-demographics). Article characteristics from this stage will be summarized descriptively and charted.

Quality assessment:

Relevant studies with extractable data and meet all eligibility criteria will undergo a quality assessment (Appendix C). Qualitative literature will be critically appraised using a modified version of two existing tools to evaluate integrity, transparency, and limitations (Critical Appraisal Skills Programme, 2018; Walsh et al., 2006). To ensure rigour in the review process, all steps will be conducted using pre-tested tools by two independent reviewers.

Review management:

To ensure rigour in the review process, all steps will be conducted using pre-tested tools by two independent reviewers. All references identified in the review will be de-duplicated with the reference management program Mendeley 1.19.8 (Elsevier Inc., New York, NY). Relevant screening, confirmation, charting, and characterization will be conducted using Microsoft Excel 2102 (Microsoft, Redmond, Washington, USA) spreadsheets.

Data extraction and analysis:

A thematic synthesis approach will be followed. Qualitative data in the form of illustrative quotes will be extracted from included studies, coded inductively, and undergo thematic synthesis to generate themes

(Harden & Thomas, 2005). Data will be analyzed using NVivo (QSR International, Melbourne, Australia) by two independent reviewers. Lastly, the Confidence in the Evidence from Reviews of Qualitative research (CERQual) approach will be used to assess how much confidence to place in each of the individual findings of the qualitative synthesis (Lewin et al., 2015).

References:

Barnoya, J., & Navas-acien, A. (2013). Protecting the world from secondhand tobacco smoke exposure: Where do we stand and where do we go from here? *Nicotine and Tobacco Research*, *15*(4), 789–804. https://doi.org/10.1093/ntr/nts200

Beck, M. J., & Hensher, D. A. (2020). Insights into the impact of COVID-19 on household travel and activities in Australia – The early days of easing restrictions. *Transport Policy*, *99*, 95–119. https://doi.org/10.1016/j.tranpol.2020.08.004

Biddlestone, M., Green, R., & Douglas, K. M. (2020). Cultural orientation, power, belief in conspiracy theories, and intentions to reduce the spread of COVID-19. *British Journal of Social Psychology*, *59*(3), 663–673. https://doi.org/10.1111/bjso.12397

Bronfenbrenner, U. (1979). *The Ecology of Human Development: Experiments by Nature and Design*. Harvard University Press. https://books.google.ca/books?hl=en&lr=&id=OCmbzWka6xUC&oi=fnd&pg=PA3&dq=The+ecology+of+human+development.+&ots=yzJVM2YLh8&sig=ej95ik7c7R4SK8sBtcEjm--V7Po#v=onepage&q=The ecology of human development.&f=false

Centers for Disease Control and Prevention. (2020). *The Social-Ecological Model: A Framework for Prevention*. https://www.cdc.gov/violenceprevention/publichealthissue/social-ecologicalmodel.html

Conner, M., & Norman, P. (2005). *Predicting health behaviour: Research and practice with social cognition models* (M. Conner & P. Norman (eds.); 2nd ed.). Open University Press.

Critical Appraisal Skills Programme. (2018). *CASP Checklist: 10 questions to help you make sense of a qualitative research*. https://casp-uk.net/wp-content/uploads/2018/01/CASP-Qualitative-Checklist-2018.pdf

Government of Canada. (2015). *Dangers of second-hand smoke*. https://www.canada.ca/en/health-canada/services/smoking-tobacco/avoid-second-hand-smoke/second-hand-smoke/dangers-second-hand-smoke.html

Harden, A., & Thomas, J. (2005). Methodological issues in combining diverse study types in systematic reviews. *International Journal of Social Research Methodology: Theory and Practice*, *8*(3), 257–271. https://doi.org/10.1080/13645570500155078

Higgins, J., & Thomas, J. (2019). *Cochrane Handbook for Systematic Reviews of Interventions: Version 6.0*. https://training.cochrane.org/handbook/current

LaRochelle-Côté, S., & Uppal, S. (2020). *Differences in the concerns of Canadians with respect to the COVID-19 pandemic*. https://www150.statcan.gc.ca/n1/en/pub/45-28-0001/2020001/article/00019-eng.pdf?st=JiBSpJAe

Lazuras, L., Rodafinos, A., Panagiotakos, D. B., Thyrian, J. R., John, U., & Polychronopoulos, E. (2009). Support for smoke-free policies in a pro-smoking culture: Findings from the European survey on tobacco control attitudes and knowledge. *International Journal of Public Health*, *54*(6), 403–408. https://doi.org/10.1007/s00038-009-0074-2

Lewin, S., Glenton, C., Munthe-Kaas, H., Carlsen, B., Colvin, C. J., Gülmezoglu, M., Noyes, J., Booth, A., Garside, R., & Rashidian, A. (2015). Using Qualitative Evidence in Decision Making for Health and Social Interventions: An Approach to Assess Confidence in Findings from Qualitative Evidence Syntheses (GRADE-CERQual). *PLOS Medicine*, *12*(10), e1001895. https://doi.org/10.1371/journal.pmed.1001895

Lumen Learning. (n.d.). *Models and Mechanisms of Public Health*. Retrieved August 25, 2020, from https://courses.lumenlearning.com/suny-buffalo-environmentalhealth/

Omura, M., Stone, T. E., Petrini, M. A., & Cao, R. (2020). Nurses’ health beliefs about paper face masks

in Japan, Australia and China: a qualitative descriptive study. *International Nursing Review*, *67*(3), 341–351. https://doi.org/10.1111/inr.12607
Thomas, J., & Harden, A. (2008). Methods for the thematic synthesis of qualitative research in systematic reviews. *BMC Medical Research Methodology*, *8*(1), 45. https://doi.org/10.1186/1471-2288-8-45
United Nations Development Programme. (2019). *Human Development Reports*. http://hdr.undp.org/en/composite/HDI
Walsh, D., Downe, S., Emden, C., al., et, Pearce, P., & Collins, L. (2006). Appraising the quality of qualitative research. *Midwifery*, *22*(2), 108–119. https://doi.org/10.1016/j.midw.2005.05.004
World Health Organization. (n.d.). *Fact sheet on smoke free legislation*. Retrieved October 14, 2020, from https://www.euro.who.int/en/health-topics/disease-prevention/tobacco/world-no-tobacco-day/2011-who-framework-convention-on-tobacco-control/fact-sheet-on-smoke-free-legislation

Appendix A: Relevance Screening Form

<table>
<tr><th>Question</th><th>Options</th><th>Definitions/additional notes</th></tr>
<tr><td>1. Does the citation describe <u>qualitative research</u> investigating <u>sociocultural and behavioural impacts</u> of <u>smoke-free or COVID-19 regulations</u> among the <u>general public</u>?</td><td>Yes
No</td><td><u>Topic:</u>
Includes the following regulatory health policies which favour disease prevention behaviours:
Smoke-free areas in public areas (e.g., parks, beaches) and where the public gather (e.g., bars, pubs, restaurants)
COVID-19 preventive measures including face coverings, mandated quarantine, stay-at-home orders, hand hygiene, and maintaining physical distancing in public spaces
Review scope only includes discussion of regulatory health policies surrounding these restrictions which apply to the entire population, related to prevention of chronic and infectious diseases.

<u>Public</u>:
Includes the general public. Exclude studies investigating (a) managers and staff about workplace health policies; (b) stakeholder perceptions about public health policies; and (c) children and youth less than 18 years old.

<u>Sociocultural and behavioural components:</u>
Includes studies investigating these as well as any other cultural, social, or psychological factors related to smoke-free policies and COVID-19. Examples: knowledge, awareness, perceptions, self-efficacy, subjective norms/social influences, motivators, facilitators, barriers, challenges, social environment constraints (e.g., lack of resources, space), cultural/religious norms, societal/social norms, trust, support, intentions, and behaviours.

<u>Include</u>
All primary qualitative research study designs, publication dates, and types, including grey literature (e.g., public opinion research).
Studies where you “can’t tell” the relevance and suspect it could be relevant.
<u>Exclude</u>
Studies on the secondary health outcomes of policies (e.g., mental health, physical activity, dietary behaviours, substance use).
Social media analyses where the age demographic of the users or posts cannot be identified
Studies which only investigate the following: institution-wide policies (e.g., prisons, military, hospitals, college campuses); policies for children (e.g., sale of tobacco, alcohol or cannabis; mandatory immunization); e-cigarettes; smoke-free homes or cars; or residential housing units
Occupational settings (e.g., restaurants) unless the general public (i.e., patrons) was interviewed</td></tr>
</table>

Appendix B: Article Characterization Form

Question	Options	Comments
1) Is this qualitative research investigating sociocultural and behavioural factors related to smoke-free or COVID-19 restrictions among the general public, published in English, French or Spanish?	Yes, qualitative study<br>Yes, mixed methods study<br>No, specify reason(s) for exclusion:<br>☐ Quantitative study<br>☐ No relevant data for extraction and synthesis<br>☐ Not relevant to review question: _________<br>☐ Other language: _________<br>☐ Not primary research<br>☐ Other:____________<br><br>If "no" is selected, submit form without proceeding further.	**General public:**<br>Includes general public who are targets for smoke-free and COVID-19 regulatory policies. **Exclude** studies interviewing stakeholders, health professionals, care providers, restaurant staff and managers. Also exclude studies in developing countries.<br><br>**Sociocultural and psychological factors:**<br>Includes various societal, cultural, social and psychological constructs that contribute to explaining an individual's behaviour. Examples include but are not limited to: attitudes; knowledge; perceived control over behaviour; self-efficacy; subjective norms/social influences; religious practices; cultural norms; motivations; facilitators; barriers; challenges; past behaviours/habits; trust; support risk perceptions/beliefs about consequences (e.g., perceived threat, susceptibility and severity); social environment constraints (e.g., resources, space).<br><br>**Qualitative research:** Aimed at understanding social phenomena, exploring issues, and answering questions of "why" and "how" as opposed to numerical summarization of results. Not usually generalizable to a whole population. Example designs include descriptive, grounded theory, phenomenology, ethnography. Examples: focus groups, interviews.<br><br>**Mixed methods research:** Conduct of qualitative and quantitative methods in the same study.<br><br>**Exclude:**<br>• Studies with no cultural, social, or individual constructs investigated<br>• Research focused on institutional settings (e.g., hospital, university campus), smoke-free homes, or residential housing units.<br>• Studies discussing mask wearing, stay-

		at-home, or physical distancing not within the context of the COVID-19 pandemic.
2) What is the publication year of this article?	_______	
3) What type of document is this article?	Journal article<br>Thesis<br>Conference paper/abstract<br>Government or research report<br>Other, please specify:_____	
4) What is the article language?	English<br>French<br>Spanish	**Exclude** studies in languages other than English, French, or Spanish
5) Where was the study conducted?<br><br>*(Check all that apply)*	North America:<br>☐ Canada<br>☐ USA<br>☐ Mexico<br>Europe:_______________<br>Australasia:_____<br>Central and South America/ Caribbean:________<br>Asia:______________________<br>Africa:_____________________<br>Not stated	Please specify the country and the continent. If the investigation was a multi-national study, please list all countries.
6) When was the study conducted?	___________<br>Not reported	Please specify year/month to year/month if available (do not extract days) [follow format example: 2000/06-2000/08]
7) What is the policy focus of this study?	Smoke-free policies:<br>☐ Restaurants<br>☐ Pubs/bars<br>☐ Parks<br>☐ Beaches<br>☐ Bus stops<br>☐ Other, specify:______<br>☐ Not specified<br>COVID-19 restrictions:<br>☐ Distancing<br>☐ Face masks<br>☐ Stay-at-home<br>☐ Hand hygiene<br>☐ Not specified	**Only specify if the study authors explicitly state their focus on one or more aspect(s).**<br><br>**Smoke-free areas =** study focuses on smoke-free policies in public, recreational, nightclub, bar/pub, and foodservice settings.<br><br>**COVID-19 restrictions =** study focuses on social/physical distancing, quarantine/lockdown, stay-at-home, and mask wearing as a result of COVID-19.
8) What is the study methodology?	Grounded theory<br>Phenomenology	**If more than one design, report ONLY study design(s) relevant to the research question.**

	Ethnography<br>Participatory action research<br>Case study<br>Other, please specify:_______<br>Not specified	**Grounded theory:** Going beyond adding to the existing body of knowledge and involves developing a new theory about a phenomenon. This new theory is grounded in data.<br><br>**Phenomenology:** Study of individuals' lived experiences of events (e.g., experience of smokers after smoke-free legislation).<br><br>**Ethnography:** Portrait of people where the study investigators tell a story or culture of a group to inform readers on the culture nuances, awareness, or sensitivity.<br><br>**Participatory action research:** Individuals and groups researching their own personal beings, socio-cultural settings, and experiences<br><br>**Case study:** In-depth investigation of a single or small number of units at a point or over a period in time (e.g., evaluation of smoke-free policies).<br><br>***Please specify the design/methodology that is identified by the author, and if none is identified explicitly then indicate "not specified".***
9) How were the data collected?	Focus groups<br>Qualitative interviews<br>Other, please specify ______<br>Not specified	
10) What was the mode of conduct?	In-person<br>Telephone<br>Virtual<br>Written response<br>Not specified	
11) Were the data collected on more than one occasion (e.g., multiple interviews implemented at different time points)?	Yes, specify number of occasions:___<br>No	E.g., for longitudinal studies

12) How were participants recruited?	Random-digit dialing<br>Marketing or survey research agency<br>Public or private database / list<br>Advertisements in public places<br>Advertisements online<br>Other, specify:_________<br>Not specified	
13) What was the final sample size?	_______________<br>Not reported	Report final sample size used for analysis. **For focus groups**, report number of groups interviewed, average number pre group, and total number of participants across all groups.
14) Was the study focused on participants with any targeted socio-demographic characteristics?	Yes, specify:<br>☐ Smokers or ex-smokers<br>☐ Migrants<br>☐ Students<br>☐ Older adults (60+)<br>☐ Low-socioeconomic status/homeless<br>☐ LGBTQ+<br>☐ Indigenous<br>☐ Other:____________<br>No / not reported	**Select options ONLY if the group was the main focus/target population of the study AS SPECIFIED BY THE AUTHORS**
15) Additional comments:	___________	

Appendix C: Quality Assessment Form

Question/quality domain	Options	Definitions/additional notes
Was there a clear statement of the research purpose/aims?	Yes<br>No	Consider the following to make a judgement:<br>• Clarity of focus, explicit purpose given, supported by prior research
Was the research design and data collection strategy clearly described and appropriate to address the research aims?	Yes<br>No	Consider the following to make a judgement:<br>• Rationale provided for research design/data collection strategy (including setting)<br>• Research design and data collection strategy were appropriate to address the research purpose/question
Was the sampling strategy clearly described and appropriate to address the research aims?	Yes<br>No	Consider the following to make a judgement:<br>• Selection criteria detailed, and description provided for how sampling was undertaken<br>• Justification for sampling strategy and selection of participants is given.
Was the method of analysis clearly described and appropriate to address the research aims?	Yes<br>No	Consider the following to make a judgement:<br>• Approach made explicit (e.g., thematic analysis, grounded theory) and described in depth<br>• Discussion of how coding systems/conceptual frameworks evolved<br>• If thematic analysis conducted, is it clear how themes were derived?
Were the findings clearly described and supported by sufficient evidence?	Yes<br>No	Consider the following to make a judgement:<br>• Did data provide sufficient depth, detail and richness? (e.g., illustrative quotes)<br>• Context described and taken into account in interpretation/results<br>• Approaches taken to ensure robustness (e.g., multiple analysts, triangulation, member checking/participant validation of results)
Was there evidence of researcher reflexivity?	Yes<br>No	Consider the following to make a judgement:<br>• Discussion of relationship between the researchers and participants during data collection<br>• Researchers' potential role and influence on study critically examined and/or discussed<br>• Evidence of how problems/complications met were dealt with

Were ethical issues taken into consideration?	Yes<br>No	Consider the following to make a judgement:<br>• Study approved by ethics committee<br>• Sufficient details provided on how the research was explained to participants and whether ethical standards were maintained<br>• Documentation of how autonomy, consent, confidentiality, anonymity were managed<br>• Documentation of any ethical dilemmas and how they were resolved
Was there evidence of study relevance and transferability?	Yes<br>No	Consider the following to make a judgement:<br>• Discussion of contribution of study to existing/prior knowledge, practice, and/or policy<br>• Areas for future research identified<br>• Limitations/weaknesses of study clearly outlined<br>• Discussion of whether or how the findings can be transferred to other populations or consideration of other ways the research may be used

Appendix D: Search Documentation

**Date(s)**	October 31, 2020 *(original search)*; December 19, 2022 *(updated)*
**Platform/Interface**	ProQuest
**Databases**	Sociological Abstracts (1952-2022)<br>Applied Social Sciences Index & Abstracts (1987-2022)<br>ProQuest Dissertations & Theses A&I (1743-2022)
**Institution**	University of Guelph
**Search string:**	("COVID-19" OR "SARS-CoV-2" OR "nCoV" OR "smoke-free" OR "tobacco-free") AND (resident* OR "older people" OR adults OR smokers OR communit* OR population* OR migrant* OR customer* OR Indigenous OR "college student*" OR "university student*") AND (enforc* OR restrict* OR policy OR policies OR bylaw OR "by-law" OR constitution* OR ban OR bans OR bann* OR regulat* OR legislation* OR mandat* OR illegal OR law OR laws OR "social distancing" OR "physical distancing" OR "wearing mask*" OR "face mask*" OR "face covering*" OR "stay-at-home" OR "shelter-in-place" OR lockdown OR quarantine OR "public health order*" OR "government order*" OR "government-issued" OR "government response*" OR cultur* OR customs OR tradition* OR religio* OR "societal norm*" OR "social norm*" OR "societal context") AND (knowledge OR awareness OR attitud* OR opinion* OR belief* OR perceive* OR perception* OR motivat* OR concern* OR challeng* OR trust OR distrust* OR support OR barrier* OR facilitator* OR intention* OR behavior* OR behaviour* OR compliance OR adhere*) AND (qualitative OR "focus groups" OR "thematic analysis" OR "semi-structured" OR "open-ended questions" OR "key informant" OR ethnograph* OR "in-depth" OR indepth OR "face-to-face" OR "mixed-methods")<br>in **Anywhere except full text - NOFT**
**Hits**	October 31, 2020 (n = 142); December 19, 2022 (n = 1098)
**Limits**	**Language:** English, French, and Spanish

**Date(s)**	October 31, 2020 *(original search)*; December 19, 2022 *(updated)*
**Platform/Interface**	APA PsycNet
**Databases**	PsycINFO (1597-2022)
**Institution**	University of Guelph
**Search string:**	("COVID-19" OR "SARS-CoV-2" OR "nCoV" OR "smoke-free" OR "tobacco-free") AND (enforc* OR restrict* OR policy OR policies OR bylaw OR "by-law" OR constitution* OR ban OR bans OR bann* OR regulat* OR legislation* OR mandat* OR illegal OR law OR laws OR "social distancing" OR "physical distancing" OR "wearing mask*" OR "face mask*" OR "face covering*" OR "stay-at-home" OR "shelter-in-place" OR lockdown OR quarantine OR "public health order*" OR "government order*" OR "government-issued" OR "government response*" OR cultur* OR customs OR tradition* OR religio* OR "societal norm*" OR "social norm*" OR "societal context") AND (knowledge OR awareness OR attitud* OR opinion* OR belief* OR perceive* OR perception* OR motivat* OR concern* OR challeng* OR trust OR distrust* OR support OR barrier* OR facilitator* OR intention* OR behavior* OR behaviour* OR compliance OR adhere*)<br>in **Any Field**
**Hits**	October 31, 2020 (n = 212); December 19, 2022 (n = 298)
**Limits**	**Age group**: Adulthood (18 yrs & older)<br>**Population group:** Human<br>**Methodology:** Qualitative study; Interview; Focus group

**Date(s)**	October 31, 2020 *(original search)*; December 19, 2022 *(updated)*
**Platform/Interface**	Web of Science
**Databases**	Web of Science Core Collection (1900-2022)
**Institution**	University of Guelph

**Search string:**	("COVID-19" OR "SARS-CoV-2" OR "nCoV" OR "smoke-free" OR "tobacco-free") AND (resident* OR "older people" OR adults OR smokers OR migrant* OR customer* OR Indigenous OR "college student*" OR "university student*") AND (enforc* OR restrict* OR policy OR policies OR bylaw OR "by-law" OR constitution* OR ban OR bans OR bann* OR regulat* OR legislation* OR mandat* OR illegal OR law OR laws OR "social distancing" OR "physical distancing" OR "wearing mask*" OR "face mask*" OR "face covering*" OR "stay-at-home" OR "shelter-in-place" OR lockdown OR quarantine OR "public health order*" OR "government order*" OR "government-issued" OR "government response*" OR cultur* OR customs OR tradition* OR religio* OR "societal norm*" OR "social norm*" OR "societal context") AND (knowledge OR awareness OR attitud* OR opinion* OR belief* OR perceive* OR perception* OR motivat* OR concern* OR challeng* OR trust OR distrust* OR support OR barrier* OR facilitator* OR intention* OR behavior* OR behaviour* OR compliance OR adhere*) AND (qualitative OR "focus groups" OR "thematic analysis" OR "semi-structured" OR "open-ended questions" OR "key informant" OR ethnograph* OR "in-depth" OR indepth OR "face-to-face" OR "mixed-methods")<br>in **Topic**
**Hits**	October 31, 2020 (n = 363); December 19, 2022 (n = 1894)
**Limits**	None

**Date(s)**	October 31, 2020 *(original search)*; December 19, 2022 *(updated)*
**Platform/Interface**	PubMed
**Databases**	PubMed (1975-2022)
**Institution**	University of Guelph
**Search string:**	("COVID-19" OR "SARS-CoV-2" OR "nCoV" OR "smoke-free" OR "tobacco-free") AND (resident* OR "older people" OR adults OR smokers OR communit* OR population* OR migrant* OR customer* OR Indigenous OR "college student*" OR "university student*") AND (enforc* OR restrict* OR policy OR policies OR bylaw OR "by-law" OR constitution* OR ban OR bans OR bann* OR regulat* OR legislation* OR mandat* OR illegal OR law OR laws OR "social distancing" OR "physical distancing" OR "wearing mask*" OR "face mask*" OR "face covering*" OR "stay-at-home" OR "shelter-in-place" OR lockdown OR quarantine OR "public health order*" OR "government order*" OR "government-issued" OR "government response*" OR cultur* OR customs OR tradition* OR religio* OR "societal norm*" OR "social norm*" OR "societal context") AND (knowledge OR awareness OR attitud* OR opinion* OR belief* OR perceive* OR perception* OR motivat* OR concern* OR challeng* OR trust OR distrust* OR support OR barrier* OR facilitator* OR intention* OR behavior* OR behaviour* OR compliance OR adhere*) AND (qualitative OR "focus groups" OR "thematic analysis" OR "semi-structured" OR "open-ended questions" OR "key informant" OR ethnograph* OR "in-depth" OR indepth OR "face-to-face" OR "mixed-methods")<br>in **Title/Abstract**
**Hits**	October 31, 2020 (n = 366); December 19, 2022 (n = 603)
**Limits**	None

Google and Google Scholar search strings:

1. COVID-19 restrictions culture beliefs report general public qualitative
2. COVID-19 orders beliefs attitudes residents qualitative
3. COVID-19 guidelines beliefs attitudes societal factors report
4. smoke-free policies community beliefs focus groups report
5. smoke-free public areas attitudes qualitative interviews
6. tobacco-free policies societal norms attitudes qualitative

Appendix E: Coding Framework for Thematic Synthesis

- ***Political environment - Facilitates behaviour change and introduces new opportunities***
  - Education and awareness increase support
  - Facilitates change in behaviour
  - Unanticipated positive outcomes
- ***Political environment - Restricts personal freedoms and highlights flaws in the system***
  - Economic concerns
  - Critiquing the state and its laws
  - Loss of freedoms
- ***Sociocultural environment - Group identity***
  - Cultural identity
  - Societal attitudes
  - Social exclusion
- ***Sociocultural environment - Social responsibility***
  - Empowers people to confront non-compliers
  - Respect and serving society
- ***Sociocultural environment - Adapting to the new normal***
  - Adjustment period
  - Disruption to routines
  - Mirroring others
- ***Physical environment – Comfort and barriers dictate compliance***
  - Context-dependent compliance
  - Comfort as a driver for behaviour
  - Original behaviour is tied to other practice/setting
