# Supplementary material for: Environmental determinants of infectious and chronic disease prevention behaviours: A systematic review and thematic synthesis of qualitative research
Source: Health Psychol Open. 2023 May 25;10(1):20551029231179157. doi: 10.1177/20551029231179157 (PMC10226319; doi:10.1177/20551029231179157)
Supplement: Supplemental Material - Environmental determinants of infectious and chronic disease prevention behaviours: A systematic review and thematic synthesis of qualitative research [file sj-pdf-3-hpo-10.1177_20551029231179157.pdf]

**CERQual Assessment – Environmental determinants of infectious and chronic disease prevention behaviours: A systematic review and thematic synthesis of qualitative research**

Description of the CERQual approach used to assess confidence in each main review finding (adapted from Lewin et al., 2015).

| Component | Explanation | Rating options |
|---|---|---|
| Adequacy of data | Determination of the degree of richness and quantity of data supporting a review finding. Includes extent that data are supported by detailed narratives and participant quotes and the number and diversity of studies and participants represented. | • No concerns<br>• Minor concerns<br>• Moderate concerns<br>• Substantial concerns |
| Relevance | The extent to which the body of evidence from the primary studies supporting the review finding is applicable to the review question context (population, phenomenon of interest, setting). | • No concerns<br>• Minor concerns<br>• Moderate concerns<br>• Substantial concerns |
| Coherence | The extent to which the review finding is well grounded in data from the contributing primary studies and provides a convincing explanation for the patterns found in these data. Judgement based on consistency of data across studies and ability to explain any contrasting or disconfirming data. | • No concerns<br>• Minor concerns<br>• Moderate concerns<br>• Substantial concerns |
| Methodological limitations | The extent to which there are problems in the design or conduct of the contributing studies. Judgement based on each study's relative contribution to the finding, the types of methodological limitations identified in the quality assessment tool and how those limitations could impact on the specific finding. | • No concerns<br>• Minor concerns<br>• Moderate concerns<br>• Substantial concerns |
| Overall assessment | Determination of overall confidence in review finding based on iterative review of each CERQual component. | • High confidence<br>• Moderate confidence<br>• Low confidence |

**S2 Table.** Summary of the overall confidence of each review finding (n = 17) identified in the thematic synthesis using the CERQual approach.

| Finding | Adequacy of data | Relevance | Coherence | Methodological limitations | Overall confidence | References |
|---|---|---|---|---|---|---|
| **Political environment – Facilitates behavior change and introduces new opportunities** | | | | | | |
| Awareness | **No concerns:** supported by 52/87 studies (53/88 articles) with rich data from most studies | **No concerns** | **Minor concerns:** some inconsistencies as to the participants' level of knowledge and support for policies | **Minor concerns:** methodological limitations in some of the represented studies | **High:** finding is supported by 52 studies with rich data, minor coherence, and methodological concerns | 1–54 |
| Facilitates behaviour change | **No concerns:** rich data from 60/87 studies (61/88 articles) | **No concerns** | **Minor concerns:** some studies reported that it was not a facilitator because the law was not respected | **Minor concerns:** methodological limitations in some of the represented studies | **High:** finding is supported by 60 studies with rich data, minor relevance, coherence, and methodological concerns | 2,3,6,7,10–12,14–27,32–35,37–39,41–43,45–53,55–76 |
| Unanticipated positive outcomes | **Minor concerns:** supported by 39/87 studies (40/88 articles), with rich data from most studies | **No concerns** | **Minor concerns:** some contrasting perspectives noted regarding benefits | **Minor concerns:** methodological limitations in some of the represented studies | **High:** finding is supported by 39 studies with limited data richness, minor coherence, and methodological concerns | 1,6–8,10,12,13,15,16,19,20,22,23,25,26,28,32,34–37,39,40,44,48,49,53,60,61,63–66,68,71,73,75,77–80 |
| **Political environment – Threat to individual rights and highlights flaws in the system** | | | | | | |
| Economic concerns | **Moderate concerns:** supported by 20/87 studies (20/88 articles), with limited depth from some studies | **No concerns** | **No concerns** | **Minor concerns:** methodological limitations in some of the represented studies | **Moderate:** finding is supported by 20 studies with mostly rich data and minor methodological concerns | 9,10,17,19,20,26,31,34,35,42,45,47,58,73,75,80–84 |
| Critiquing the state and its laws | **No concerns:** rich data from 53/87 studies (53/88 articles) | **No concerns** | **Minor concerns:** participants' perspectives of the law varied slightly depending on time of policy implementation | **Minor concerns:** methodological limitations in some of the represented studies | **High:** finding is supported by 53 studies with rich data, minor coherence, and methodological concerns | 2–7,11,12,14,17–19,21,24–27,32,35,37,40–43,45–47,51–58,61,62,64,66–71,73,76,77,80,84–87 |
| Loss of freedoms | **Minor concerns:** supported by 33/87 studies (33/88 | **No concerns** | **Minor concerns:** Non-smokers did not agree with this | **Minor concerns:** methodological limitations in some of | **High:** finding is supported by 33 studies with mostly | 2,3,6,12,14,18,21,24,26–29,32,40,41,44,48,53,54,56–58,60,62,68,70,73,76–78,82,83,87 |

| Finding | Adequacy of data | Relevance | Coherence | Methodological limitations | Overall confidence | References |
|---|---|---|---|---|---|---|
| | articles), with limited depth of data provided in some studies | | finding because it did not negatively affect them much | the represented studies | rich data and minor coherence and methodological concerns | |
| **Sociocultural environment - Group formation** | | | | | | |
| Cultural identity | **Minor concerns:** supported by rich data from 34/87 studies (35/88 articles) | **No concerns** | **No concerns** | **Minor concerns:** methodological limitations in some of the represented studies | **Moderate:** finding is supported by 34 studies with rich data, and minor methodological concerns | 1,2,4–6,9–11,14,18,21,23,25,26,29–32,34,37,39,41–43,49,50,52,54,57,60,62,63,68,83,84,88 |
| Societal attitudes | **Minor concerns:** supported by rich data from 51/87 studies (52/88 articles) | **No concerns** | **Minor concerns:** some societies had contrasting norms | **Minor concerns:** methodological limitations in some of the represented studies | **High:** finding is supported by 51 studies with rich data, minor relevance, and methodological concerns | 1,2,17,18,21,23,25,26,28–31,3,32,34,35,38–43,45,4,46,48–52,54,60–62,9,63–65,68,69,71,73,76,78,79,10,87,88,11,14–16 |
| Social exclusion | **Moderate concerns:** rich data from 33/87 studies (34/88 articles) | **No concerns** | **No concerns:** participants' views on stigma were similar but the behavioural response was different depending on the community | **Minor concerns:** methodological limitations in some of the represented studies | **Moderate:** finding is supported by 33 studies with rich data, moderate relevance concerns and minor methodological concerns | 1,2,5,6,12–15,17,18,21,23,25,26,28–30,32,37,38,41,45,49,54,55,57,62–64,68,69,71,76,78,88 |
| **Sociocultural environment - Social responsibility** | | | | | | |
| Empowerment | **Moderate concerns:** rich data from 31/87 studies (31/88 articles) | **No concerns** | **No concerns** | **Minor concerns:** methodological limitations in some of the represented studies | **Moderate:** finding is supported by 31 studies with rich data and minor methodological concerns | 2,5,6,8,10,12,14,16,17,21,22,25,26,29–31,41,48,50,51,54,56,57,59,61–65,68,88 |
| Respect and serving society | **Minor concerns:** supported by 50/87 studies (51/88 articles), with rich data in most studies | **No concerns** | **No concerns** | **Minor concerns:** methodological limitations in some of the represented studies | **High:** finding is supported by 50 studies with rich data and minor methodological concerns | 1,2,4,6–19,21,23–26,28–31,34,37–40,42–46,49,51–54,56,59,60,64,66,68,71,73,76,78,85 |
| **Sociocultural environment - Adapting to the new normal** | | | | | | |

| Finding | Adequacy of data | Relevance | Coherence | Methodological limitations | Overall confidence | References |
|---|---|---|---|---|---|---|
| Adjustment period | **No concerns:** rich data from 54/87 studies (55/88 articles) | **No concerns** | **No concerns** | **Minor concerns:** methodological limitations in some of the represented studies | **High:** finding is supported by 54 studies with rich data and minor methodological concerns | 7,8,10,12–20,22,23,25,26,28,30,32–40,42,43,45,48–53,55–58,60–63,65,68,69,71,73,76,77,79,80,85,86 |
| Lifestyle disruption | **No concerns:** rich data from 46/87 studies (46/88 articles) | **No concerns** | **No concerns** | **Minor concerns:** methodological limitations in some of the represented studies | **High:** finding is supported by 46 studies with rich data and minor methodological concerns | 4,7,8,10,12,15,18–22,24–27,32,34–37,40,42,46,48,50–53,56,59,61,62,64,66,71,73,75–78,80,82,85–87 |
| Mirroring others | **Substantial concerns:** supported by data from 17/87 studies (18/88 articles), but limited depth in several studies | **Moderate concerns:** no perspectives provided from non-smokers and only some coverage from the COVID-19 context | **No concerns** | **Minor concerns:** methodological limitations in some of the represented studies | **Low:** finding is supported by 17 studies with limited data richness, moderate relevance concerns, and minor methodological concerns | 5,6,10,21,23,25,26,30,39,43,45,48,49,51,54,63,69,71,73 |
| **Physical environment – Comfort and barriers dictate compliance** | | | | | | |
| Context-dependent compliance | **Substantial concerns:** data supported by 23/87 studies (23/88 articles), but limited data depth in some studies | **Minor concerns:** weak evidence from COVID-19 studies | **No concerns** | **Minor concerns:** methodological limitations in some of the represented studies | **Moderate:** finding is supported by 23 studies with limited data richness, and minor methodological concerns | 2,3,5,10,12,14,17,21,24,26,37,40,41,43,45,51,52,56–58,64,69,85 |
| Comfort as a driver for behaviour | **Minor concerns:** supported by 45/87 studies (46/88 articles), with rich data provided in most studies | **No concerns** | **Minor concerns:** Participants' feelings on comfort and discomfort varied | **Minor concerns:** methodological limitations in some of the represented studies | **Moderate:** finding is supported by 45 studies with rich data, minor relevance concerns, minor coherence and methodological concerns | 2,4,6,11,13,14,16,17,19,21,23,24,26,28,30–33,35,37,41–45,48,49,51,54,57–59,61,63–68,71,73,76,78,80,87,88 |
| Original behaviour is tied to other practice/setting | **Moderate concerns:** supported by 22/87 studies (23/88 articles), with rich data | **Minor concerns:** data not supported across many COVID-19 studies and non- | **No concerns** | **Minor concerns:** methodological limitations in some of the represented studies | **Moderate:** finding is supported by 22 studies with rich data, minor relevance, and | 5,6,11,12,23,26,28,30–32,37,39,49,51,54,58,63–66,71,73,83,88 |

| Finding | Adequacy of data | Relevance | Coherence | Methodological limitations | Overall confidence | References |
|---|---|---|---|---|---|---|
| | provided in most studies | smokers not experiencing this issue | | | methodological concerns | |

## References


1. Zhang YSD, Noels KA, Young-Leslie H, et al. "Responsible" or "strange?" Differences in face mask attitudes and use between Chinese and non-East Asian Canadians during COVID-19's first wave. *Front Psychol* 2022; 13: 1061.

2. Fearnley L, Wu X. Beyond Asian 'mask culture': Understanding the ethics of face masks during the Covid-19 pandemic in Singapore. *Crit Public Health*. Epub ahead of print 2022. DOI: 10.1080/09581596.2022.2114315.

3. Williams SN, Armitage CJ, Tampe T, et al. Public perceptions of non-adherence to pandemic protection measures by self and others: A study of COVID-19 in the United Kingdom. *PLoS One* 2021; 16: e0258781.

4. Lee CT, Kanji R, Wang AH, et al. Cultural contexts during a pandemic: A qualitative description of cultural factors that shape protective behaviours in the Chinese-Canadian community. *BMC Public Health* 2021; 21: 1–11.

5. Benincasa L. Greek university students and the smoke-free law: Learning about rights and duties in a community of practice. *Qual Rep* 2019; 24: 1399–1422.

6. Betzner AE, Boyle RG, Luxenberg MG, et al. Experience of smokers and recent quitters with smokefree regulations and quitting. *Am J Prev Med* 2012; 43: S163–S170.

7. Brooke J, Clark M. Older people's early experience of household isolation and social distancing during COVID-19. *J Clin Nurs* 2020; 29: 4387–4402.

8. Chen AT, Ge S, Cho S, et al. Reactions to COVID-19, information and technology use, and social connectedness among older adults with pre-frailty and frailty. *Geriatr Nurs (Minneap)* 2021; 42: 188–195.

9. Chief C, Sabo S, Clark H, et al. Breathing clean air is Są'áh Naagháí Bik'eh Hózhóó (SNBH): A culturally centred approach to understanding commercial smoke-free policy among the Diné (Navajo People). *Tob Control* 2016; 25: i19–i25.

10. Hassan SM, Ring A, Tahir N, et al. The impact of COVID-19 social distancing and isolation recommendations for Muslim communities in North West England. *BMC Public Health* 2021; 21: 1–11.

11. Hassan SM, Ring A, Tahir N, et al. How do Muslim community members perceive COVID-19 risk reduction recommendations - A UK qualitative study? *BMC Public Health* 2021; 21: 1–14.

12. Kamin T, Perger N, Debevec L, et al. Alone in a time of pandemic: Solo-living women coping with physical isolation. *Qual Health Res* 2021; 31: 203–217.

13. Polivka BJ, Eldeirawi K, Huntington-Moskos L, et al. Mask use experiences, COVID-19, and adults with asthma: A mixed-methods approach. *J Allergy Clin Immunol Pract* 2022; 10: 116–123.

14. Koon AD, Mendenhall E, Eich L, et al. A spectrum of (Dis)Belief: Coronavirus frames in a rural midwestern town in the United States. *Soc Sci Med*; 272. Epub ahead of print 1 March 2021. DOI: 10.1016/J.SOCSCIMED.2021.113743.

15. Lohiniva AL, Dub T, Hagberg L, et al. Learning about COVID-19-related stigma, quarantine and isolation experiences in Finland. *PLoS One*; 16. Epub ahead of print 1 April 2021. DOI: 10.1371/JOURNAL.PONE.0247962.

16. Mollborn S, Mercer KH, Edwards-Capen T. "Everything is connected": Health lifestyles and

teenagers' social distancing behaviors in the COVID-19 pandemic. *Sociol Perspect* 2021; 64: 920–938.

17. Moss SM, Sandbakken EM. 'Everybody needs to do their part, so we can get this under control.' Reactions to the Norwegian government meta-narratives on COVID-19 measures. *Polit Psychol* 2021; 42: 881–898.

18. Nilsson G, Ekstam L, Axmon A, et al. Old overnight: Experiences of age-based recommendations in response to the COVID-19 pandemic in Sweden. *J Aging Soc Policy* 2021; 33: 359–379.

19. Rhodes SD, Mann-Jackson L, Alonzo J, et al. A rapid qualitative assessment of the impact of the COVID-19 pandemic on a racially/ethnically diverse sample of gay, bisexual, and other men who have sex with men living with HIV in the US South. *AIDS Behav* 2021; 25: 58–67.

20. Günther-Bel C, Vilaregut A, Carratala E, et al. A mixed-method study of individual, couple, and parental functioning during the state-regulated COVID-19 lockdown in Spain. *Fam Process* 2020; 59: 1060–1079.

21. Shelus VS, Frank SC, Lazard AJ, et al. Motivations and barriers for the use of face coverings during the COVID-19 pandemic: Messaging insights from focus groups. *Int J Environ Res Public Health* 2020; 17: 1–12.

22. Sweet L, Bradfield Z, Vasilevski V, et al. Becoming a mother in the 'new' social world in Australia during the first wave of the COVID-19 pandemic. *Midwifery*; 98. Epub ahead of print 1 July 2021. DOI: 10.1016/J.MIDW.2021.102996.

23. Hackshaw LE. *The implications of smoke-free legislation for NHS stop smoking services*. University of Bath (United Kingdom), http://opus.bath.ac.uk/23357/ (2010).

24. Van Alboom M, Baert F, Wauters A, et al. When, why, and how do people deviate from physical distancing measures during the COVID-19 pandemic: A mixed-methods study. *Psychol Belg* 2021; 61: 262–273.

25. Wang Q, Liu JKK, Walsh CA. Identities: Experiences and impacts of the COVID-19 pandemic from the perspectives of older Chinese immigrants in Canada. *China J Soc Work* 2021; 14: 153–171.

26. Hargreaves K, Highet G. *Evaluation of smokefree England: A longitudinal, qualitative study*, https://phrc.lshtm.ac.uk/assets/uploads/files/PHRC_A5_06_Final_Report.pdf (2009, accessed 3 November 2020).

27. Zwain A. George Town Heritage City traditional shophouses residents respond to COVID-19 pandemic: Issues and possible solutions. *J Hum Behav Soc Environ* 2021; 31: 394–408.

28. Heim D, Ross A, Eadie D, et al. Public health or social impacts? A qualitative analysis of attitudes toward the smoke-free legislation in Scotland. *Nicotine Tob Res Off J Soc Res Nicotine Tob* 2009; 11: 1424–1430.

29. Helweg-Larsen M, Tobias MR, Cerban BM. Risk perception and moralization among smokers in the USA and Denmark: A qualitative approach. *Br J Health Psychol* 2010; 15: 871–886.

30. Highet G, Ritchie D, Platt S, et al. The re-shaping of the life-world: Male British Bangladeshi smokers and the English smoke-free legislation. *Ethn Health* 2011; 16: 519–533.

31. Bottorff JL, Carey J, Mowatt R, et al. Bingo halls and smoking: Perspectives of First Nations women. *Health Place* 2009; 15: 1014–1021.

32. Van der Heiden S, Gebhardt WA, Willemsen MC, et al. Behavioural and psychological responses of lower educated smokers to the smoke-free legislation in Dutch hospitality venues: A qualitative study. *Psychol Health* 2013; 28: 49–66.

33. Juszczyk D, Gillison F. Comparing and contrasting responses to tobacco control and obesity policies: A qualitative study. *Public Health Nutr* 2019; 22: 927–935.

34. Cecilia Johnson M, Saletti-Cuesta L, Tumas N. Emotions, concerns and reflections regarding the COVID-19 pandemic in Argentina. *Cien Saude Colet* 2020; 25: 2447–2456.

35. Tay YL, Abdullah Z, Chelladorai K, et al. Perception of the movement control order during the COVID-19 pandemic: A qualitative study in Malaysia. *Int J Environ Res Public Health* 2021; 18: 8778.

36. Levkovich I, Shinan-Altman S. Impact of the COVID-19 pandemic on stress and emotional reactions in Israel: A mixed-methods study. *Int Health* 2021; 13: 358–366.

37. Lock K, Adams E, Pilkington P, et al. Evaluating social and behavioural impacts of English smoke-free legislation in different ethnic and age groups: Implications for reducing smoking-related health inequalities. *Tob Control* 2010; 19: 391–397.

38. McCloy CA, Keller-Olaman S, Schwartz R, et al. *Tobacco-free sports and recreation policies: Evaluation of policy implementation in hockey settings*. Toronto, https://otru.org/wp-content/uploads/2012/12/OTRU_SER_2012.pdf (October 2012, accessed 21 December 2022).

39. Williams RJ. *Assessing how to increase smokers' motivation to quit*. University of Hawaii at Manoa, http://hdl.handle.net/10125/101786 (2011, accessed 21 December 2022).

40. Williams SN, Armitage CJ, Tampe T, et al. Public perceptions and experiences of social distancing and social isolation during the COVID-19 pandemic: A UK-based focus group study. *BMJ Open* 2020; 10: e039334.

41. Louka P, Maguire M, Evans P, et al. 'I think that it's a pain in the ass that I have to stand outside in the cold and have a cigarette': Representations of smoking and experiences of disapproval in UK and Greek smokers. *J Health Psychol* 2006; 11: 441–451.

42. Alqahtani MMJ, Arnout BA, Fadhel FH, et al. Risk perceptions of COVID-19 and its impact on precautionary behavior: A qualitative study. *Patient Educ Couns* 2021; 104: 1860–1867.

43. Benham JL, Lang R, Kovacs Burns K, et al. Attitudes, current behaviours and barriers to public health measures that reduce COVID-19 transmission: A qualitative study to inform public health messaging. *PLoS One* 2021; 16: e0246941.

44. Bozdağ F. The psychological effects of staying home due to the COVID-19 pandemic. *J Gen Psychol* 2021; 148: 226–248.

45. DeJonckheere M, Waselewski M, Amaro X, et al. Views on COVID-19 and use of face coverings among U.S. youth. *J Adolesc Heal* 2021; 68: 873–881.

46. Vilme H, Akin-Odanye EO, Sauls DL, et al. A social-ecological exploration of college and university students' COVID-19 infection preventive behaviors. *Am J Heal Educ* 2022; 53: 256–265.

47. Enria L, Waterlow N, Rogers NT, et al. Trust and transparency in times of crisis: Results from an online survey during the first wave (April 2020) of the COVID-19 epidemic in the UK. *PLoS One*; 16. Epub ahead of print 1 February 2021. DOI: 10.1371/JOURNAL.PONE.0239247.

48. Fristedt S, Carlsson G, Kylén M, et al. Changes in daily life and wellbeing in adults, 70 years and older, in the beginning of the COVID-19 pandemic. *Scand J Occup Ther* 2021; 1–11.

49. Hackshaw L, Bauld L, McEwen A. Stop smoking service clients' views following the introduction of smoke-free legislation in England. *J Smok Cessat* 2012; 7: 47–54.

50. He L, Traphagan JW. A preliminary exploration of attitudes about COVID-19 among a group of older people in Iwate Prefecture, Japan. *J Cross Cult Gerontol* 2021; 36: 1–19.

51. Burton A, McKinlay A, Dawes J, et al. Understanding barriers and facilitators to compliance with UK social distancing guidelines during the COVID-19 pandemic: A qualitative interview study. *Behav Chang* 2022; 1–21.

52. Eraso Y, Hills S. Intentional and unintentional non-adherence to social distancing measures during COVID-19: A mixed-methods analysis. *PLoS One* 2021; 16: :e0256495.

53. Gazibara T, Maksimovic N, Dotlic J, et al. Experiences and aftermath of the COVID-19 lockdown among community-dwelling older people in Serbia: A qualitative study. *J Eval Clin Pract* 2022; 28: 631–640.

54. Schönweitz F, Eichinger J, Kuiper JML, et al. The social meanings of artifacts: Face masks in the COVID-19 pandemic. *Front Public Heal* 2022; 10: 789.

55. Haire B, Gilbert GL, Kaldor JM, et al. Experiences of risk in Australian hotel quarantine: A qualitative study. *BMC Public Health* 2022; 22: 1–8.

56. Leather JZ, Keyworth C, Epton T, et al. "We want to live a little longer and our family want[s] us around": A summative content analysis of adherence to COVID-19-related guidelines using the Theoretical Domains Framework. *Br J Health Psychol* 2022; 27: 1119–1152.

57. Bell K, McCullough L, Salmon A, et al. 'Every space is claimed': Smokers' experiences of tobacco denormalisation. *Sociol Health Illn* 2010; 32: 914–929.

58. Berg CJ, Lessard L, Parelkar PP, et al. College student reactions to smoking bans in public, on campus and at home. *Health Educ Res* 2011; 26: 106–118.

59. Kim J, Kim Y, Ha J. Changes in daily life during the COVID-19 pandemic among South Korean older adults with chronic diseases: A qualitative study. *Int J Environ Res Public Health*; 18. Epub ahead of print 1 July 2021. DOI: 10.3390/IJERPH18136781.

60. Moyce S, Velazquez M, Claudio D, et al. Exploring a rural Latino community's perception of the COVID-19 pandemic. *Ethn Health* 2021; 26: 126–138.

61. Takashima R, Onishi R, Saeki K, et al. Perception of COVID-19 restrictions on daily life among Japanese older adults: A qualitative focus group study. *Healthcare* 2020; 8: 450.

62. Vanhamel J, Meudec M, Van Landeghem E, et al. Understanding how communities respond to COVID-19: Experiences from the Orthodox Jewish communities of Antwerp city. *Int J Equity Health* 2021; 20: 1–13.

63. Hargreaves K, Amos A, Highet G, et al. The social context of change in tobacco consumption following the introduction of 'smokefree' England legislation: A qualitative, longitudinal study. *Soc Sci Med* 2010; 71: 459–466.

64. Kaufman P, Griffin K, Cohen J, et al. Smoking in urban outdoor public places: Behaviour, experiences, and implications for public health. *Health Place* 2010; 16: 961–968.

65. González-Salgado I de L, Rivera-Navarro J, Sureda X, et al. Qualitative examination of the perceived effects of a comprehensive smoke-free law according to neighborhood socioeconomic status in a large city. *SSM - Popul Heal* 2020; 11: 100597.

66. Kelly BC. Smoke-free air policy: Subcultural shifts and secondary health effects among club-going young adults. *Sociol Health Illn* 2009; 31: 569–582.

67. Lee JK, Lin L, Lim MJR, et al. National tobacco control policies from the perspectives of Singapore young male adults. *J Psychoactive Drugs* 2020; 52: 5–12.

68. Li J, Collins D. Smoking environments in transition: The experiences of recent Chinese migrants to Canada. *Health Soc Care Community* 2017; 25: 65–74.

69. Parnell A, Box E, Biagioni N, et al. Attitudinal and behavioural responses to increasing tobacco control regulation among high smoking prevalence groups: A qualitative study. *Drug Alcohol Rev* 2019; 38: 92–100.

70. Reiter PL, Wewers ME, Paskett ED, et al. Ohio Appalachian residents' views on smoke-free laws and cigarette warning labels. *Rural Remote Health* 2012; 12: 1945.

71. Ritchie D, Amos A, Martin C. Public places after smoke-free - A qualitative exploration of the changes in smoking behaviour. *Health Place* 2010; 16: 461–469.

72. Vijayaraghavan M, Olsen P, Weeks J, et al. Older African American homeless-experienced smokers' attitudes toward tobacco control policies—Results from the HOPE HOME Study. *Am J Heal Promot* 2018; 32: 381–391.

73. Wakefield M, Cameron M, Murphy M. Potential for smoke-free policies in social venues to prevent smoking uptake and reduce relapse: A qualitative study. *Health Promot Pract* 2009; 10: 119–127.

74. Widome R, Joseph AM, Polusny MA, et al. Talking to Iraq and Afghanistan war veterans about tobacco use. *Nicotine Tob Res* 2011; 13: 623–626.

75. Ares G, Bove I, Vidal L, et al. The experience of social distancing for families with children and adolescents during the coronavirus (COVID-19) pandemic in Uruguay: Difficulties and opportunities. *Child Youth Serv Rev*; 121. Epub ahead of print 1 February 2021. DOI: 10.1016/J.CHILDYOUTH.2020.105906.

76. Falvo I, Zufferey MC, Albanese E, et al. Lived experiences of older adults during the first COVID-19 lockdown: A qualitative study. *PLoS One* 2021; 16: e0252101.

77. Kotwal AA, Holt-Lunstad J, Newmark RL, et al. Social isolation and loneliness among San Francisco Bay Area older adults during the COVID-19 shelter-in-place orders. *J Am Geriatr Soc* 2021; 69: 20–29.

78. Ritchie D, Amos A, Martin C. 'But it just has that sort of feel about it, a leper'--Stigma, smoke-free legislation and public health. *Nicotine Tob Res* 2010; 12: 622–629.

79. Satterlund TD, Lee JP, Moore RS. Changes in smoking-related norms in bars resulting from California's Smoke-Free Workplace Act. *J Drug Educ* 2012; 42: 315–326.

80. Adams LM, Gell NM, Hoffman E V., et al. Impact of COVID-19 'stay home, stay healthy' orders on function among older adults participating in a community-based, behavioral intervention study. *J Aging Health* 2021; 33: 458–468.

81. Bryant L, Damarin AK, Marshall Z. Tobacco control recommendations identified by LGBT

Atlantans in a community-based participatory research project. *Prog Community Heal Partnerships Res Educ Action* 2014; 8: 269–279.

82. White S, Baird W. Disadvantaged former miners' perspectives on smoking cessation: A qualitative study. *Health Educ J* 2013; 72: 755–760.

83. Crosbie E, Snider KM, McMillen R, et al. Expanding smoke-free communities: Attitudes and beliefs surrounding smoke-free casinos and bars in Washoe County, Nevada. *J Hosp Manag Heal Policy* 2020; 4: 23–23.

84. Satterlund T, Antin T, Lee J, et al. Cultural factors related to smoking in San Francisco's Irish bars. *J Drug Educ* 2009; 39: 181–193.

85. Power N, Warmelink L, Wallace R. Prosocial rule breaking, ingroups and social norms: Parental decision-making about COVID-19 rule breaking in the UK. *J Community Appl Soc Psychol*; 1. Epub ahead of print 2022. DOI: 10.1002/CASP.2650.

86. Heid AR, Cartwright F, Wilson-Genderson M, et al. Challenges experienced by older people during the initial months of the COVID-19 pandemic. *Gerontologist* 2021; 61: 48–58.

87. Pederson A, Okoli CT, Hemsing N, et al. Smoking on the margins: A comprehensive analysis of a municipal outdoor smoke-free policy. *BMC Public Health* 2016; 16: 1–13.

88. Tan QH. Smoking spaces as enabling spaces of wellbeing. *Health Place* 2013; 24: 173–182.
